# Energy evolution and crack development characteristics of sandstone under freeze-thaw cycles by digital image correlation

**DOI:** 10.1371/journal.pone.0283378

**Published:** 2023-04-20

**Authors:** Junzu Ma, Jiaxu Jin, Jiaju Feng, Zhifa Qin

**Affiliations:** School of Civil Engineering, Liaoning Technical University, Fuxin, Liaoning, China; Semnan University, ISLAMIC REPUBLIC OF IRAN

## Abstract

Freeze-thaw erosion is the main reason for rock mass instability in cold regions and poses major threats to public safety. In this study, the stress threshold, energy, and strain field evolution of sandstone and the variation in stress intensity factor of fractures in various stress fields were all investigated after freeze-thaw cycles by uniaxial compression tests and digital image correlation technology. The results show that the elastic modulus, crack initiation stress, and peak stress all fell by *97%*, *92*.*5%*, and *89*.*9%*, respectively, as the number of freeze-thaw cycles approaches 80. Elastic energy’s storage capacity also dropped from *0*.*85* to *0*.*17*. Sandstone’s strain was increased by freeze-thaw erosion, which also improved ductility and shortened the cracking time. The stress intensity factor at the crack tip was positively correlated with the tip inclination angle and negatively correlated with the number of freeze-thaw cycles. This study provides a useful reference for understanding the stability of rock masses and the characteristics of crack derivation in cold regions.

## Introduction

China is one of the countries with the largest distribution of cold regions in the world, whose frozen soil area accounts for 75% of the total land area of the country. With the development of the Belt and Road economic belt, project construction in cold region is progressively increasing. Due to the large temperature difference in the cold region, the interior of rock mass was damaged by the freeze-thaw cycle of water molecules, which would cause progressive failure of the rock mass [1, 2]. Therefore, in order to ensure the stability and safety of construction, the progressive failure behavior of rock mass after freeze-thaw erosion was studied. Freeze-thaw erosion is actually the result of the continuous development of cracks due to the volume expansion of water-ice phase transition at low temperature, and the development of cracks will lead to the decrease of rock mass strength. Li et al. [3] performed uniaxial compression tests on granite with freeze-thaw cycles, and found that the mass of granite increased and the uniaxial compressive strength decreased after freeze-thaw cycles. Mei et al. [4] studied the static and dynamic mechanical properties of freeze-thaw eroded red sandstone using uniaxial compression and Hopkinson pressure bar tests, and concluded that the static and dynamic strengths of freeze-thawed specimens were significantly lower than those of unfreeze-thawed ones. The physical and mechanical parameters of rocks can directly reflect the failure degree of freeze-thawed rocks, but if the change of physical and mechanical properties is the only consideration, the process of rock failure would be ignored.

The evolution of energy was accompanied by the progressive failure of rocks [5]. Therefore, the failure process can be more accurately analyzed from the perspective of energy. Tang et al. [6] analyzed the causes of rock failure by using the principle of energy conversion and found that the sudden release of stored elastic energy could led to the failure of rock mass units. In order to study the law of energy change of rock under different unloading levels, Luo et al. [7] introduced proportional energy relation to quantify elastic energy and dissipated energy. Fan et al. [8] studied the energy evolution mechanism of sandstone with different freeze-thaw cycles and found that the total energy, elastic strain energy and dissipative energy all decreased with the increase of freeze-thaw cycles.

The progressive failure process of rocks can also be studied by analyzing the variation of strain field on rock surface. Methods of recording strain field on rock surface include sticking a strain gauge, digital image processing, etc. [9, 10]. As a non-contact method to measure the change of material mesoscopic spatial structure, digital image processing has been widely used in meso-structure analysis of asphalt concrete, soil and rock in recent 20 years. Ma et al. [11] quantitatively analyzed the strain field of rocks by means of digital speckle processing, and divided the rock failure into three stages: uniform distribution stage, local failure stage and overall failure stage. Li et al. [12] analyzed the failure mode of rock mass affected by compression-shear effect based on the strain nephogram obtained by digital speckle processing. Yang et al. [13] used digital speckle system to quantitatively analyze the displacement field of rock crack location under impact loading effect.

The majority of the research mentioned above focuses on the energy and strain field evolution of sandstone, which does not adequately reflect how sandstone transforms from brittleness to ductility after freeze-thaw cycles and the evolution of fracture features. As a result, this study examined the connection between the brittleness of sandstone and elastic energy. It also used a digital image correlation technique to study the strain field of sandstone, the crack evolution law, and the alteration of the stress intensity factor at the crack tip under various freeze-thaw cycles.

## Research method

### Specimen descriptions and preparation

**Fig 1:**
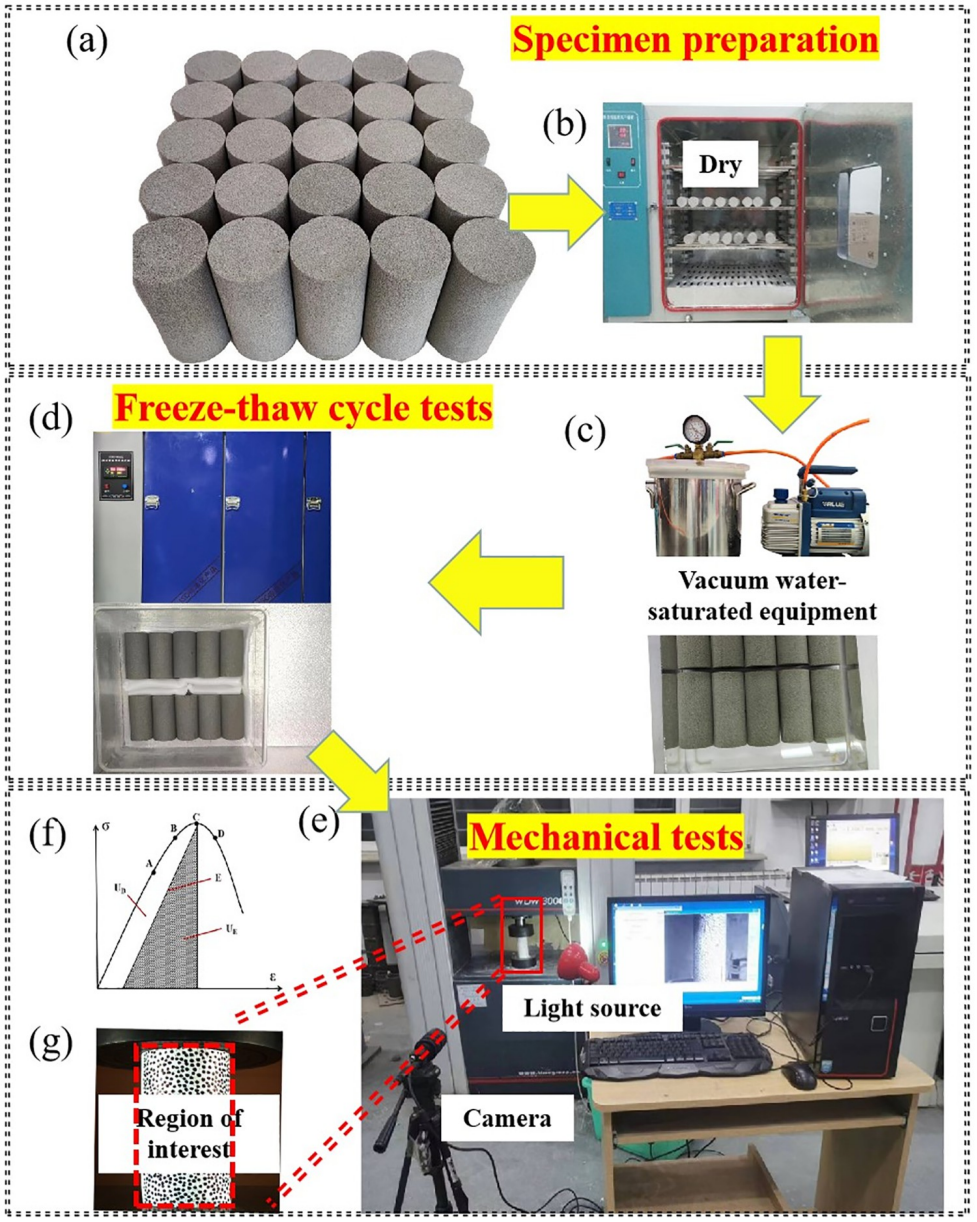
Test equipment and procedure. (a) Preparation of rock specimens (b) Electric drying oven (c) Vacuum water-saturation equipment (d) Freeze-thaw cycle equipment (e) Digital speckle and mechanical test (f) Energy evolution characteristics (g) Speckle specimen.

Sandstone specimen adopts a kind of sedimentary rock in western Sichuan plateau. It is sound-proof, moisture-absorbing and radiation-free, and thus usually used as building material. The uniaxial compressive strength is about *40 GPa*. The elasticity modulus is about *3*.*8 GPa*. The Poisson’s ratio is *0*.*25* and the density is *2*.*4 g/cm*^*3*^. All the specimens came from the same rock mass. It was made into cylindrical specimens with a diameter of *50 mm* and a length of *100 mm*, and the end surfaces of specimens were polished to ensure its flatness is less than *0*.*05 mm* (Fig 1A), according to International Society for Rock Mechanics (ISRM) Standard. A temperature of 105°C was used to bake the sample (Fig 1B), and it was weighed every *12h*. The sample was considered to have fully dried when the difference in weight reading was less than *0*.*01 g*. The sample was then placed in a vacuum water-saturated device (Fig 1C) for water-saturated treatment.

According to the variation of annual average temperature under natural conditions in this area, the freezing temperature of sandstone specimen was set to *-20°C*, and the melting temperature was set to *20°C*. The time of freezing and thawing was set to *12h*, and the same type of sandstone was subjected to *20*, *40*, *60*, *80* freeze-thaw cycles respectively. The freeze-thaw cycle is shown in the Fig 1D.

Load-displacement data can be directly obtained during the experiment. However, it is difficult to measure the change of local strain field of the specimen. Due to the small specimen size, sticking a strain gauge will affect the stability and accuracy of measurement. So non-contact monitoring equipment is needed for monitoring the specimen. In this study, the digital speckle system was adopted as the detection device for local strain field. The digital speckle method needs to add artificial speckle on the surface of the specimen so that the local displacement field on the surface of the specimen could be obtained. To increase the contrast ratio of the pattern, firstly, matte white paint was sprayed on the specimen surface as primer and then the black mark pen was used to draw black speckles. The process followed the principle of uniform and random distribution (Fig 1G).

### Compression instrument and DIC system

Fig 1E showed the schematic diagram of the compression instrument and DIC system. In the test, the load-displacement data of the specimen during compression was obtained by the processing system of the compression instrument, and the displacement nephogram was obtained by processing the image from DIC measurement system.

The universal testing machine for servo control system was used in the test. The maximum axial load was *300 KN*. Its pressure chamber was made up of base and pressure head, which could process the cylindrical specimen with a diameter of *50 mm*. The strain field of specimen surface under uniaxial compression was observed by DIC technique.

Digital image correlation technology (DIC) was composed of a CMOS (Complementary metaloxidee semiconductor) camera placed on the beam of tripods, and a LED light providing stable light source. Photos taken by this camera could be processed by software. Artificial speckles were added to the surface of the specimen, and the strain field of the surface was obtained by DIC.

### Experimental process and parameter recorder

The specimen was loaded by the strain control method with a constant axial displacement decline rate of *0*.*05 mm/min*. The data after the peak of the stress curve can be accurately captured by the controlled strain loading method. The F-T cycles were *20、40、60、80* respectively. The test was conducted three times under each testing circumstance, and average values were calculated. At the beginning of the test, the compression instrument and the digital speckle system started recording synchronously with a time interval of *1s*. The detailed procedures are presented as follows:

1. Draw speckles on specimens coated with white matte primer.
2. Place the speckled specimen in pressure chamber.
3. Adjust the light and position of the digital camera properly.
4. Adjust DIC system parameters according to specimen size.
5. Carry out the testing and image-processing.

### Data and digital image processing

#### Energy evolution

Rock failure is a combination of energy accumulated by elastic strain and the dissipation of stress propagation. The energy evolution law could be obtained through the stress-strain curve of the specimen without considering the thermal energy loss caused by friction between particles [14]. The total energy of sandstone specimen at peak strength includes elastic energy and dissipative energy, as shown in Fig 1F and Eq (1) [15].

$$U=U_{E}+U_{D}$$ (1)

Where *U* is the total energy output from the compression instrument to sandstone; *U*_*E*_ is the elastic potential energy at peak stress; *U*_*D*_ is the dissipative energy generated by plastic deformation, which is not recoverable [16]. When the slope of stress-strain curve approximates elastic modulus *E*, the triangular area of the shadow part can represent the elastic strain energy *U*_*E*_ [17]. The area of the enclosed region bounded by the vertical line and the stress-strain curve is known as the total energy *U*. This enclosed area could be calculated by integration.

#### Image processing

DIC analyzes the displacement field by comparing the speckle images of the sample before and after deformation. The relationship between reference image and deformed image in DIC system is expressed as [18]:

$$\varphi_{2}\left(\bar{x}+\bar{d}(\bar{x};\bar{a})\right)=\varphi_{1}\left(\bar{x}\right)$$ (2)

Where *φ*_2_ is the intensity of pixel $\bar{x}$ in reference image and $\varphi_{1}\left(\bar{x}\right)$ the intensity of pixel $\bar{x}$ in deformed image, respectively. $\bar{d}(\bar{x};\bar{a})={\left[u_{x},u_{y}\right]}^{T}$ is the displacement vector, *u*_*x*_ and *u*_*y*_ are the displacement along *x* and *y* directions, respectively. $\bar{a}$ represents the parameters to determine the displacement vector in a given model.

When calculating displacement, the reference image is firstly partitioned into blocks (could be overlapped), then the displacement parameters $\bar{a}$ is resolved for each block by solving Eq (2) with a optimization format. Generally, the objective function is often constructed by maximizing the correlation coefficient of the image block before and after deformation, as expressed in Eq (3).

$$C(a)=\frac{\sum_{\bar{x}\in A}\left[\left[\varphi_{1}(\bar{\text{x}})-{\bar{\varphi }}_{1}]\cdot [\varphi_{2}\left(\bar{x}+\bar{d}(\bar{x};\bar{a})\right)-{\bar{\varphi }}_{2}\right]\right]}{{\left[\sum_{\bar{x}\in A}{\left[\varphi_{1}(\bar{x})-{\bar{\varphi }}_{1}\right]}^{2}\cdot {\sum_{\bar{x}\in A}\left[\varphi_{2}(\bar{\text{x}}+\bar{d}(\bar{x};\bar{a}))-{\bar{\varphi }}_{2}\right]}^{2}\right]}^{\frac{1}{2}}}$$ (3)

where *C*(*a*) represents the calculated image block, ${\bar{\varphi }}_{1}$ and ${\bar{\varphi }}_{2}$ are the average of *φ*_1_ and *φ*_2_ in *C*(*a*), respectively. When displacement field is obtained, the strain field could be calculated using some numerical differential methods.

## Results and discussion

### Mechanical properties of sandstone under freeze-thaw cycle

**Fig 2:**
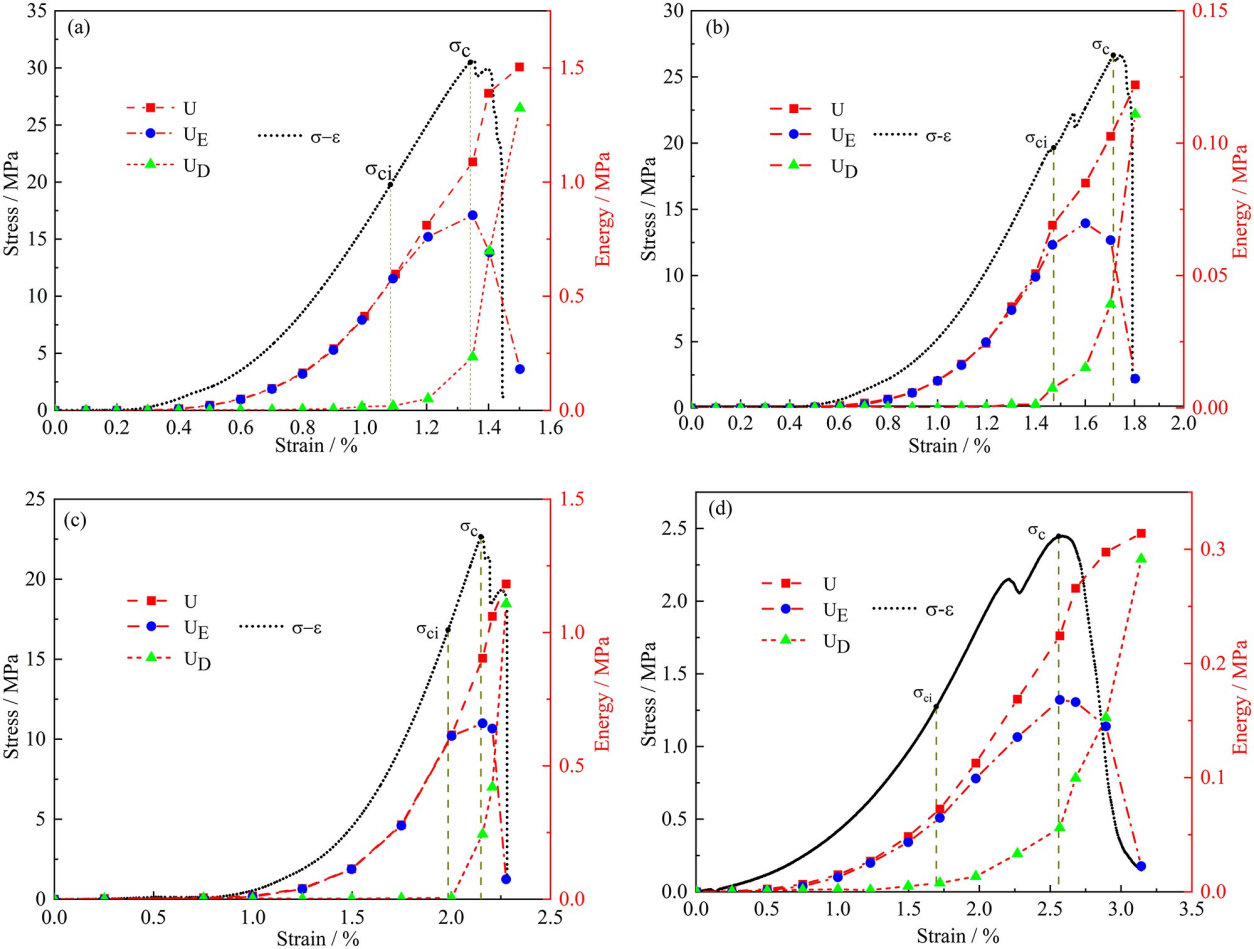
Stress-strain curve and energy evolution law of sandstone with different freeze-thaw cycles. (a) *N = 20* (b) *N = 40* (c) *N = 60* (d) *N = 80*.

The stress-strain curves of sandstone subjected to different freeze-thaw cycles were shown in Fig 2. It could be found in the stress-strain diagram obtained from the uniaxial compression test that the curve had obvious characteristics in different stages. During the first stage of loading, with the increase of strain, the stress changes fluctuated, during which there was ups and downs due to the uneven distribution of pores within the sandstone. At this time, the specimen was in the compaction stage, which made the sandstone structure more compact. The curve of the second stage rose linearly, and the sandstone particles at this time were regarded as completely elastic materials. The deformation of sandstone at this stage was elastic deformation. In the third stage, the slope of the curve decreased gradually with the increase of strain, and the shape of the curve was convex. This stage was the most active stage of crack development within the specimen. Macroscopic cracks were gradually formed on the surface of the specimen with the loading of stress. In the fourth stage, the sandstone was destroyed and the curve showed a downward trend, but the specimens with different freeze-thaw cycles had different characteristics of decline, and the slope of the curve decreased gradually with the increase of freeze-thaw cycles.

Table 1 showed the changes of mechanical properties and stress thresholds of three sandstone specimens in each group. Table 2 showed the standard deviation of each group of data. Elastic modulus was the most basic mechanical property of sandstone, which represented the ability of specimen to resist deformation [19]. The initial elastic modulus of the specimen was *3*.*8 GPa*. As the freeze-thaw cycle test progressed, the elastic modulus of the specimens after *20*, *40*, *60* and *80* freeze-thaw cycles decreased by about *62%*, *65%*, *77%* and *97%*, respectively. With the increase of freeze-thaw cycles, the elastic modulus of sandstone specimens decreased, and it was close to complete failure when freeze-thaw cycles reached *80* times.

**Table 1:** Physical and mechanical parameters of sandstone with different freeze-thaw cycles.

Freeze-thaw cycles /*N*	Elastic modulus /*GPa*			Initiation stress *σ*_*ci*_ /*MPa*			Peak stress *σ*_*c*_/*MPa*			*σ*_*ci*_*/σ*_*c*_ %		
	Specimen											
	*1*	*2*	*3*	*1*	*2*	*3*	*1*	*2*	*3*	*1*	*2*	*3*
*20*	*1*.*46*	*1*.*53*	*1*.*49*	*25*.*15*	*23*.*78*	*24*.*57*	*30*.*60*	*33*.*28*	*31*.*35*	*82*.*19*	*71*.*45*	*78*.*37*
*40*	*1*.*32*	*1*.*28*	*1*.*35*	*17*.*51*	*18*.*23*	*17*.*33*	*26*.*73*	*27*.*59*	*29*.*31*	*65*.*51*	*66*.*07*	*59*.*13*
*60*	*0*.*88*	*1*.*20*	*0*.*91*	*17*.*13*	*17*.*65*	*16*.*28*	*22*.*54*	*23*.*67*	*21*.*89*	*75*.*99*	*74*.*57*	*74*.*37*
*80*	*0*.*13*	*0*.*25*	*0*.*19*	*1*.*34*	*2*.*56*	*1*.*59*	*2*.*44*	*3*.*67*	*3*.*59*	*54*.*92*	*69*.*75*	*44*.*29*

**Table 2:** The standard deviation of each group of data.

Freeze-thaw cycles /*N*	Standard deviation		
	Elastic modulus /*GPa*	Initiation stress *σ*_*ci*_ /*MPa*	Peak stress *σ*_*c*_ /*MPa*
*20*	*0*.*035*	*0*.*688*	*1*.*383*
*40*	*0*.*035*	*0*.*676*	*1*.*314*
*60*	*0*.*077*	*0*.*692*	*0*.*901*
*80*	*0*.*060*	*0*.*644*	*0*.*688*

**Fig 3:**
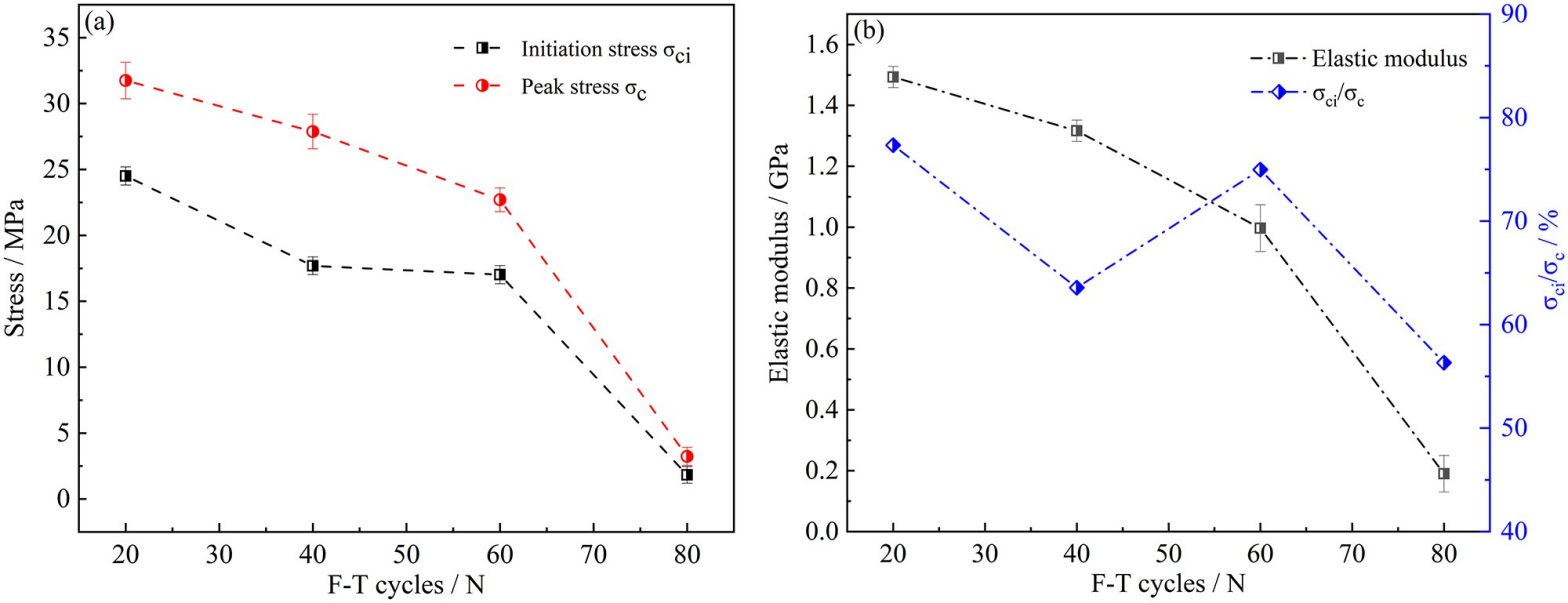
The stress threshold of sandstone specimens under different conditions.

The stress threshold of sandstone specimens under different conditions would be different in compression stage [20]. As shown in Fig 3, with the increased of freeze-thaw cycles, the initiation stress and peak stress decreased. The average value of crack initiation stress decreased from *24*.*5 MPa* (*N = 20*) to *1*.*8 MPa* (*N = 80*), which was reduced by *92*.*5%*. The average value of peak stress decreased from *31*.*6 MPa* (*N = 20*) to *3*.*2 MPa* (*N = 80*), which was reduced by *89*.*9%*. The decreasing values of the two were basically similar, but the ratio of initiation stress to peak stress gradually decreased with the increase of freeze-thaw cycles, from *77*.*3%* (*N = 20*) to *56*.*3%* (*N = 80*). However, it was discovered that the curve of *σ*_*ci*_*/σ*_*c*_ exhibited an increasing tendency once there had been *60* freeze-thaw cycles. This was due to the fact that as the number of freeze-thaw cycles reached *40*, the sandstone’s pores broke into microscopic fractures, which caused *σ*_*c*_ to sharply decline. The sample’s interior fissures fully developed as the freeze-thaw process progressed, and the skeleton structure tended to be stable. At this moment, *σ*_*ci*_*/σ*_*c*_ would continue to decrease. Chen et al. [21] believed that during the freeze-thaw cycle of sandstone, the shear stress could accelerate the cracking and propagation of cracks, so the crack initiation stress was greatly reduced, resulting in a change in the stress threshold of sandstone. Taheri et al. [22] believed that the change of rock stress threshold was closely related to the damage inside the rock, and the damage caused by freeze-thaw erosion was also the reason for weakening the stress threshold.

### Effect of freeze-thaw cycles on energy evolution of sandstone

During the compression process, the cracks within the sandstone continuously initiated, fractured and linked up. There was energy conversion in the process of crack evolution. In order to obtain the energy evolution law during uniaxial compression, the energy-strain curve was added to the stress-strain curve.

Fig 2 showed the energy evolution trend of sandstone specimens with different freeze-thaw cycles. The evolution of total energy, elastic energy and dissipative energy was less affected by freeze-thaw cycles. The total energy of the sandstone increased nonlinearly. In the pre-peak stage of the stress-strain curve, the elastic energy showed an upward trend, the growth rate was the fastest in the elastic stage, and the elastic energy gradually decreased in the post-peak stage of the stress-strain curve. The dissipation energy always increased in a nonlinear way, and the growth rate reached its highest when the sample reached its peak stress.

The main driving force of rock failure was elastic potential energy [23]. The peak value of the elastic energy curve represented the energy storage capacity of the rock, and the storage of elastic energy could be used to resist the failure caused by external load. The ability of rocks to store elastic energy was related to the stress state of rocks and the cohesive force between mineral particles. Erosion on sandstone specimens by freeze-thaw cycles would reduce the cohesive force among mineral particles. With the increase of freeze-thaw cycles, the energy storage capacity of sandstone specimens gradually decreased. The elastic energy peaks of specimens with *20*, *40*, *60* and *80* freeze-thaw cycles were *0*.*85 MPa*, *0*.*70 MPa*, *0*.*65 MPa* and *0*.*17 MPa*, respectively. When freeze-thaw cycles reached *80*, the energy storage capacity decreased significantly. The slope of the elastic energy decline stage was called the elastic energy release rate. As freeze-thaw cycles increased, the elastic energy release rate gradually slowed down, from *7*.*13* (*N = 20*) to *0*.*413* (*N = 80*). In the experiment, it was discovered that the sandstone sample with *20* freeze-thaw cycles achieved the peak stress and failed instantly with a severe effect, showing that the brittleness of sandstone was quite great at this time. Gu et al. [24] proposed the concept of a three-dimensional stress coefficient. This study found that rocks with large energy storage would have a dramatic impact when they were unstable. The proportion of the yield stage of the sample’s stress-strain curve grew after the number of freeze-thaw cycles reached *80*, and when failure occurred, the process of crack expansion on the sample’s surface was gradual, showing that the sandstone’s ductility was quite great at this time. The elastic energy that has built up inside the sample is what mostly contributes to its failure. Because there was greater elastic energy inside sandstone with high freeze-thaw durations, it was more severely affected. Accordingly, this study held that the more elastic energy present during the compression stage, the more brittle the sandstone was. Wang et al. [25] found that the damage inside the rock determined the ability of energy storage by establishing a theoretical model of elasticity and plasticity. At the same time, the combination of elastic energy accumulation and energy dissipation also caused damage inside the rock. As a result, the alternating energy shifted, and the newly created damage in the sandstone following freeze-thaw cycles interacted with one another. Based on the relationship between the energy dissipation process and the failure, Wang et al. [26] proposed a new brittleness index by considering rock failure. Therefore, this study suggested that the brittleness of rock could be studied from the perspective of elastic energy.

**Fig 4:**
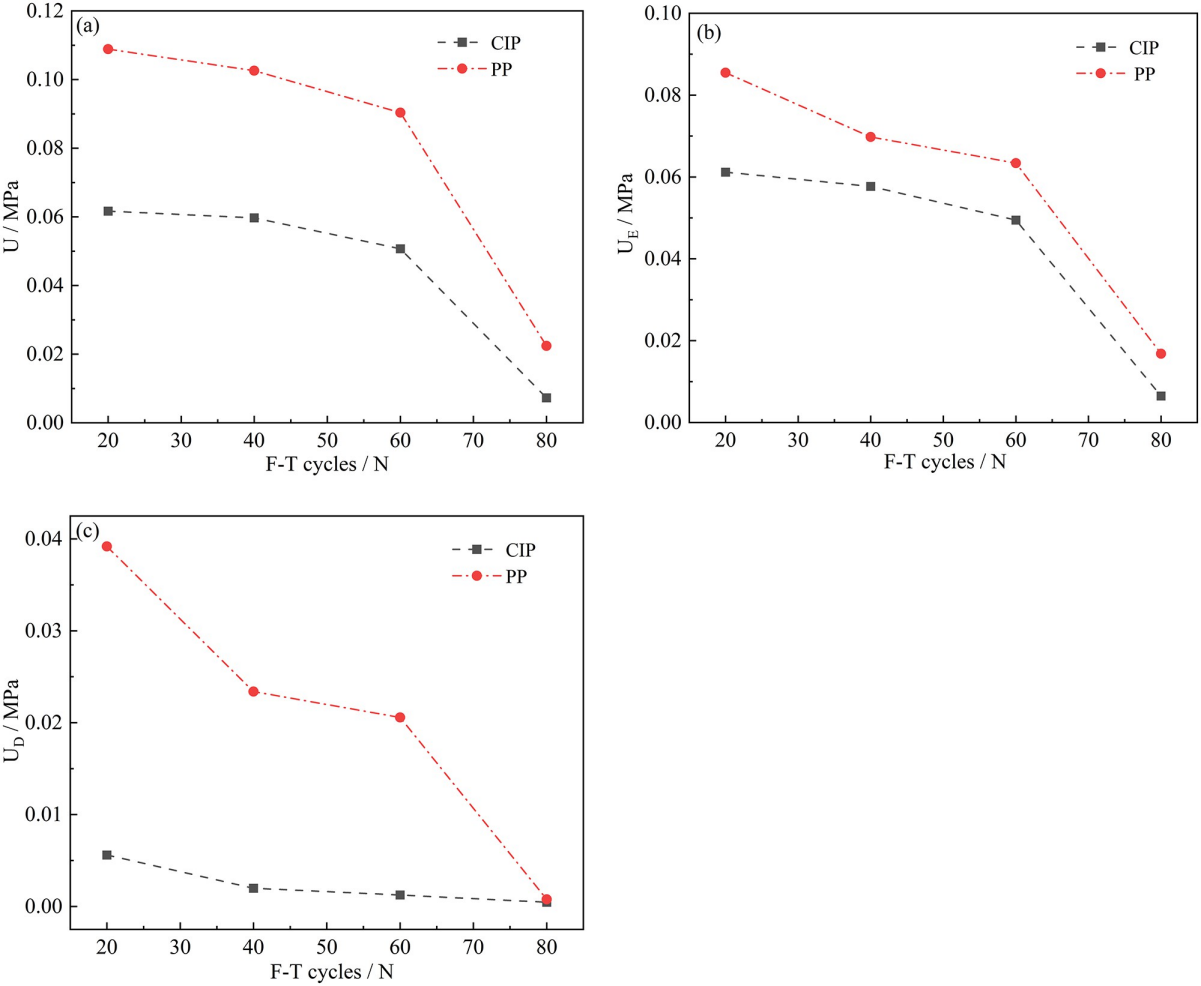
F-T effects on energy at various characteristic points. (a) Total energy (b) Elastic energy (c) Dissipative energy.

The energy at various stress thresholds decreased as the number of freeze-thaw cycles increased, but the rate of decline varied (Fig 4). CIP represents Crack initiation Point. PP represents Peak Point. Except for the dissipation energy curve at the fracture initiation stress point, each curve had clear inflection points once the number of freeze-thaw cycles reached *60*. Because of the frost heave effect, the crack was in a tensile state. The dissipation energy would not have a clear inflection point at the crack initiation stress point at this time because the elastic energy in the crack was large. The lower dissipation would not appear to have an obvious inflection point at the crack initiation stress point. Ma et al. [27] found that the energy distribution parameters of soft rock were significantly affected by freeze-thaw cycles. Hou et al. [28] found that the fracture toughness and energy release rate of sandstone gradually slowed down with the increased number of freeze-thaw cycles.

### Local strain field and crack evolution characteristics of sandstone with different freeze-thaw cycles

In order to study the erosion of different parts of sandstone specimen caused by freeze-thaw cycles, this study showed the field strain evolution process of specimen with freeze-thaw cycles of *20* to *80*. Point A (*σ*_*ci*_), point B (pre-peak *90% σ*_*c*_), point C (*σ*_*c*_) and point D (post-peak *90% σ*_*c*_) were the positions of the selected feature points on the complete stress-strain curve (Fig 1F).

**Fig 5:**
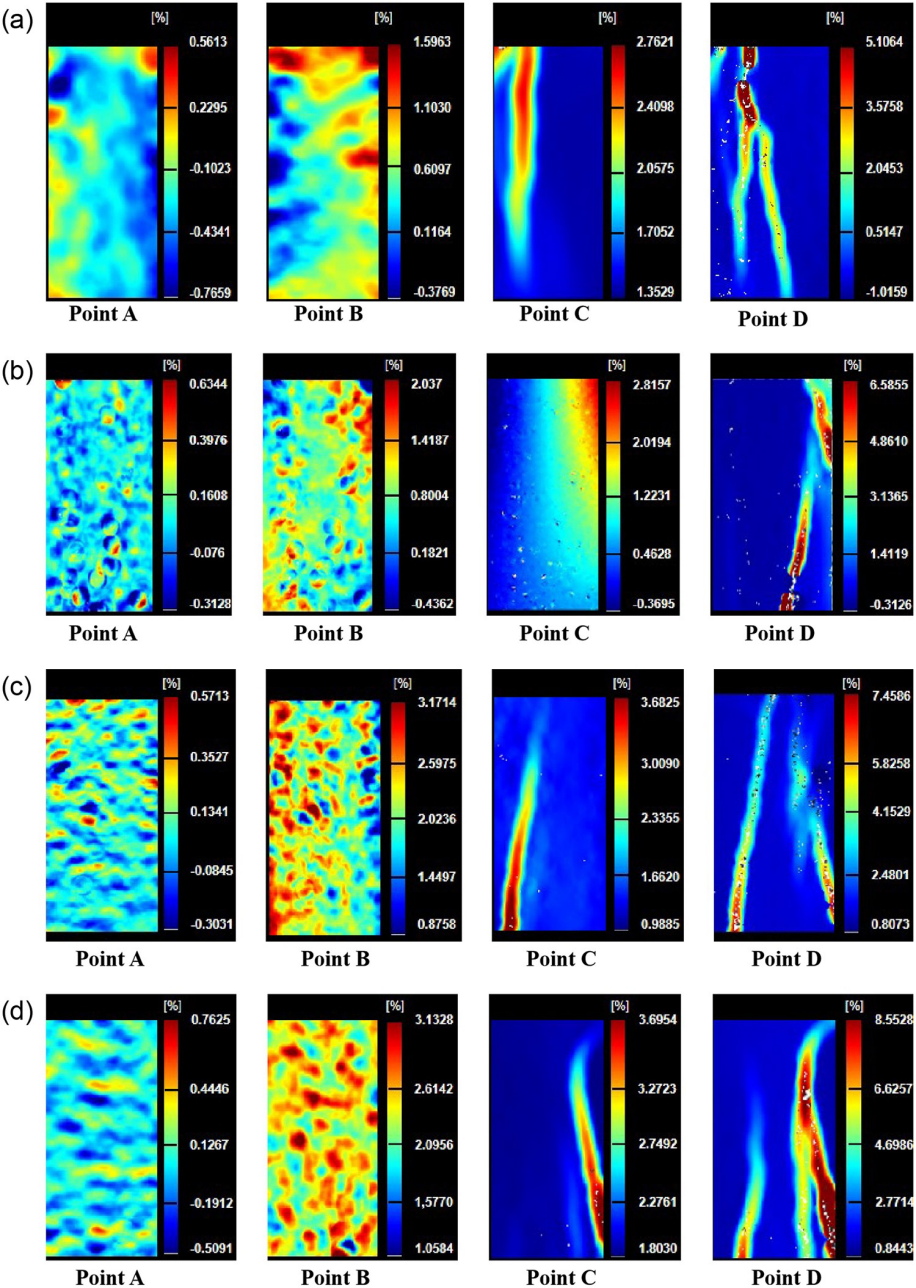
Local strain field evolution of sandstone with different freeze-thaw cycles. (a) *N = 20* (b) *N = 40* (c) *N = 60* (d) *N = 80*.

Fig 5 showed the evolution process of the principal strain field of sandstone specimen with different freeze-thaw cycles under uniaxial compression conditions obtained by digital image correlation technology (DIC). In different loading stages, the strain field on the specimen surface had different characteristics. At point A in the pre-peak stage, the main strain field was uniformly distributed, and the color of the nephogram was close to the monochromatic pattern, but there were also some unique color areas on the surface of the sample. In the image captured at point B of the stress-strain curve, areas with unique and obvious colors began to extend on the original basis, and new areas with unique colors were continuously generated at other positions on the sample surface. At the point C of the stress-strain curve, many small areas with similar directions produced strains that converge on the surface of the sample, and the outline of the main crack was completely presented, and as the loading progressed, the main crack continued to expand. At the D point of the stress-strain curve, the main crack had reached the maximum degree of expansion and extension, and the surface of the specimen had developed derived cracks. The characteristics of the strain field at point B of the stress-strain curve of the specimen with different freeze-thaw cycles were different. As freeze-thaw cycles increased, the area of significant color region also increased. Moreover, the development modes of derived cracks of specimens with different freeze-thaw cycles were different. The derived cracks of the specimens with freeze-thaw cycles of *20* and *40* were new fine textures that branch out while the main crack extended (Fig 5A and 5B). As the loading progressed, it gradually developed and expanded. The derived cracks developed from the specimen with freeze-thaw cycles of *60* and *80* have been separated from the main cracks, and there was a certain distance between the starting points of the two cracks. And the development direction of the two cracks was not parallel, if the specimen was long enough, the two cracks would intersect at one point (Fig 5C and 5D).

The cracks formed by the sandstone specimens with less freeze-thaw cycles were instantaneous, and the crack tip propagation width was small. The failure type belonged to brittle failure. With the increase of the freeze-thaw cycles, the formation rate of cracks was relatively slow. The tip width of the surface crack of the specimen increased gradually during the extension process. The failure type belonged to ductile failure. According to the study of Yahaghi et al. [29] the failure modes of sandstone failure surfaces with different freeze-thaw cycles were different. As the freeze-thaw cycles increased, the failure surface was closer to the ’Z’ shape. Cao et al. [30] thought that freeze-thaw cycles would weaken the toughness of sandstone, which was also the reason why failure type of sandstone changed from brittle failure to ductile failure.

### Strain characteristics of cracked area and non-cracked area

**Fig 6:**
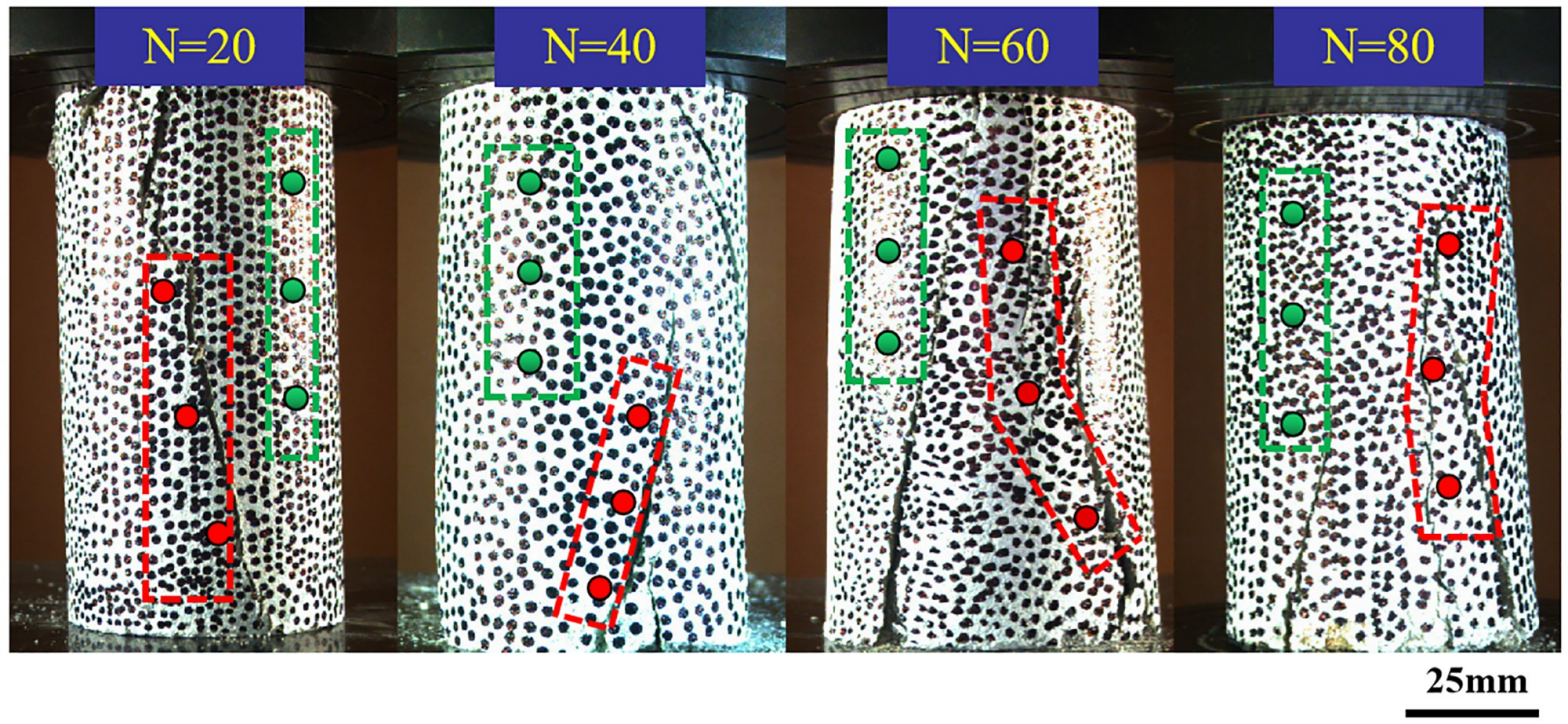
Location of monitoring points in the cracked zone and the non-cracked zone.

Sandstone is affected by freeze-thaw cycles, and strain concentration will be formed in different areas of its surface. After these strain concentrations converge, a path will be opened for the derivation of cracks. In this paper, the area where the main crack located was defined as the cracked area, and the area parallel to the main crack and without obvious cracks was defined as the non-cracked area. In order to quantitatively analyze the strain field of cracked area and non-cracked area, three monitoring points were set up in cracked area and non-cracked area respectively. The red point is the monitoring point of the cracked zone; Green point is the monitoring point of non-cracked zone, as shown in Fig 6.

**Fig 7:**
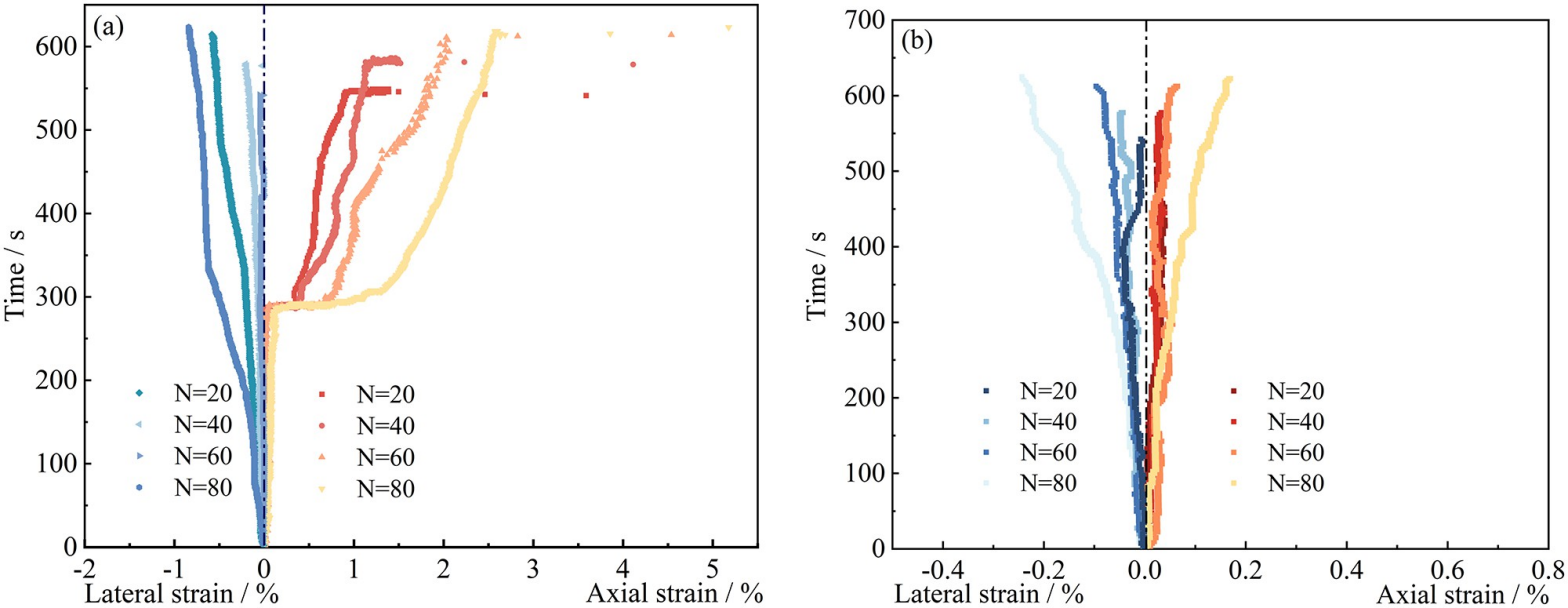
The variation of strain with time. (a) Strain in cracked area (b) Strain in non-cracked area.

By calculating the average field strain (including Radial strain and Axial strain) of the three points, the influence of freeze-thaw cycles on the strain field of specimen surface was obtained. Fig 7 showed the average local strain of sand samples with different freeze-thaw times.

As shown in Fig 7A, the local strain in the cracked area was obvious at about *294 s* for the sample with *20* freeze-thaw cycles, and with the increase of freeze-thaw cycles, the significant local axial strain appeared earlier. When the number of freeze-thaw cycles reached *80*, the sample produced significant local axial strain at about *176 s*. When the progressive failure inside the specimen accumulated to a certain extent, significant local axial strain began to appear on the surface of the specimen. As the loading progressed, the strain on the monitoring point in the cracked area gradually increased. The specimen with *20* freeze-thaw cycles was mainly composed of tensile cracks. With the increased of freeze-thaw cycles, the monitoring points around the cracked area showed an obvious shear slip phenomenon. The radial strain of the cracked area increased with the increase of freeze-thaw cycles, and the growth trend was close to linear growth.

In Fig 7B, the change of the local strain at the monitoring points in the non-cracked area was close to linear with the increase of the loading time, but there was a slight mutation on the curve, indicating that there were also small cracks in the non-cracked area. With the increase of freeze-thaw cycles, the strain at the monitoring points in the non-cracked area also increased gradually; the axial strain increased from *0*.*04* (*N = 20*) to *0*.*16* (*N = 80*), and the radial strain increased from *-0*.*05* (*N = 20*) to *-0*.*24* (*N = 80*). The strain change in the non-cracked area was not as significant as that in the cracked area, but it was also affected by the freeze-thaw cycle. The bonding force between sandstone particles was weakened by hydration and freeze-thaw erosion, resulting in greater strain on sandstone samples. As the number of freeze-thaw cycles increased, the time required for the cracked area to produce significant local axial strain was shorter, which indicated that freeze-thaw erosion would reduce the strength of the sample. The higher the number of freeze-thaw cycles, the faster the sample would crack [31, 32]. The strain in the cracked zone and the non-cracked zone increased with the increase in the number of freeze-thaw cycles. The strain at the monitoring point of the sample experiencing high freeze-thaw cycles was larger, and the time required for the sample to fail was longer. Although the non-cracked area wasn’t entirely destroyed, there were many tiny variations in the strain curve, as shown in Fig 7B, suggesting that many micro-cracks would be produced. The number of micro-cracks increased with the number of freeze-thaw cycles [33, 34]. When studying the influence of frost heaving force on cracks, Huang et al. found that the new frost heaving crack started at the tip of the crack and extended in the direction of the original fracture surface [35].

### Effect of freeze-thaw cycles on the stress intensity factor

**Fig 8:**
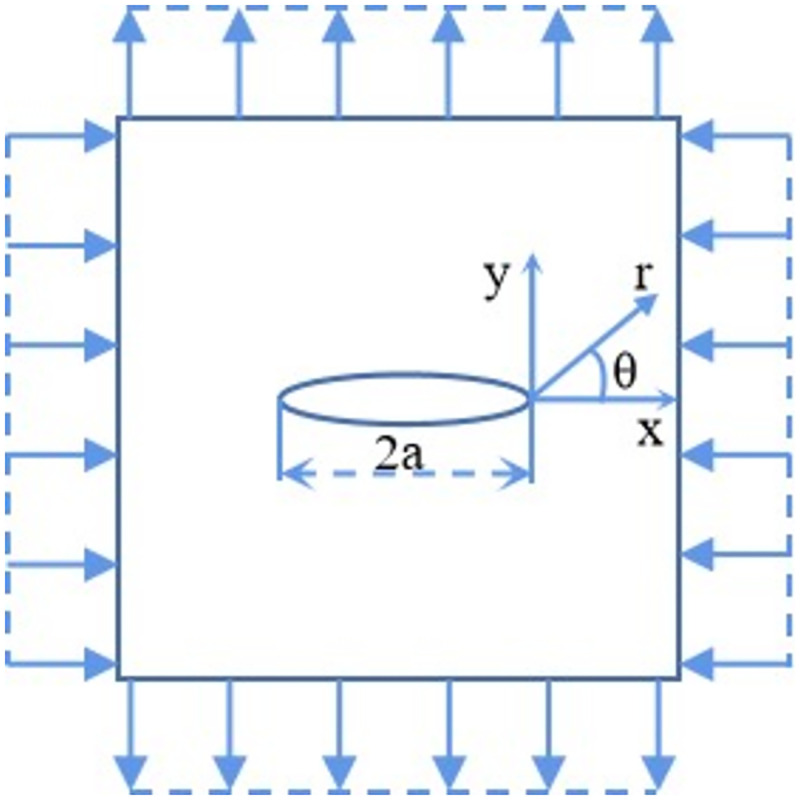
The fracture characteristics of rock units.

Sandstone has numerous micro and macro cracks. These cracks are type I cracks and are formed by tensile forces since sandstone is a brittle material. Freeze-thaw erosion causes the fracture initiation stress to fluctuate, but because the crack tip radius *r* tends to *0*, the stress surrounding the tip is almost infinite, making it impossible for the stress field to accurately define the stress intensity at the crack tip. The notion of stress intensity factor in fracture mechanics theory is provided for study in order to examine the impact of freeze-thaw erosion on crack derivation, as shown in the formula (4) and (5). Sandstone units with various freeze-thaw times have initial cracks. The initial crack tip is the coordinate origin, *r* is the radial component of the crack tip radius, and is the circumferential component of the crack, as shown in Fig 8.

The Eqs (4) and (5) for type I stress intensity factor *K*_*1*_ is as follows [36]:

$$u_{\text{x}}=\frac{K_{1}}{2\mu }\sqrt{\frac{\text{r}}{2\pi }}\text{cos}\frac{\theta }{2}\left(k-1+2{\text{sin}}^{2}\frac{\theta }{2}\right)$$ (4)

$$u_{\text{y}}=\frac{K_{1}}{2\mu }\sqrt{\frac{\text{r}}{2\pi }}\text{sin}\frac{\theta }{2}\left(k-1+2{\text{cos}}^{2}\frac{\theta }{2}\right)$$ (5)

Where *K*_*1*_ represents the stress intensity factor, *θ* represents the dip angle of crack tip. $k=\left\{\begin{array}{c} \left(3-\mu )/(1+\mu \right)\\ 3-4\mu \end{array}\right.$.

**Fig 9:**
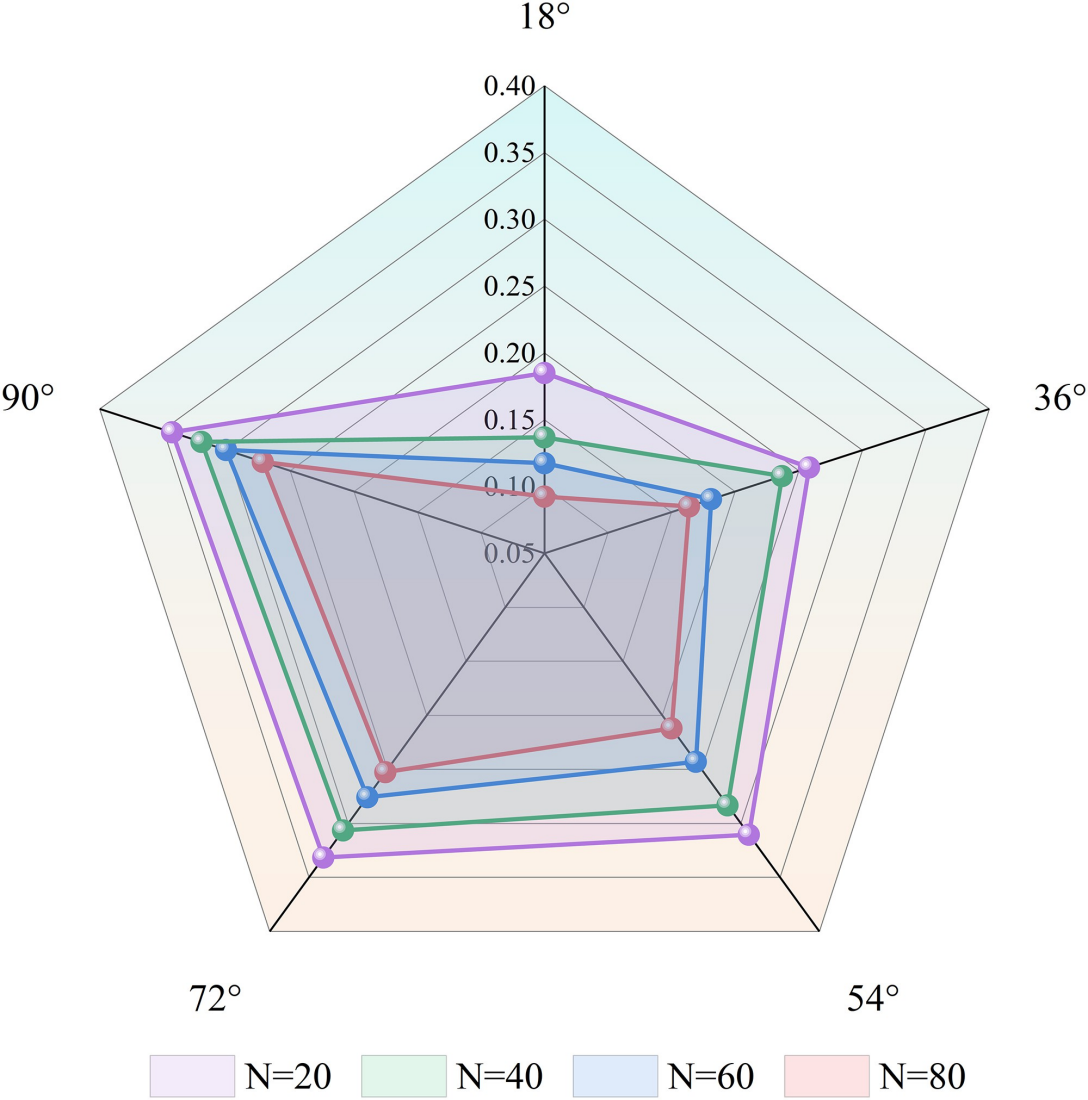
The variation law of the stress intensity factor at the crack tip.

The stress intensity factor of the sandstone type I crack tip is extracted from the displacement field of *200* pixels, and the change in the stress intensity factor with the quantity of freeze-thaw cycles is obtained, as shown in Fig 9. Under the same freeze-thaw cycle conditions, the stress intensity factor at the crack tip gradually increased with increasing crack dip angle. The stress intensity factor at the crack tip decreased as the number of freeze-thaw cycles increased. The tension near the crack tip and the crack length were all positively connected with the stress intensity factor. Generally, with the increased number of freeze-thaw cycles, the length of microcracks in sandstone will be longer, which will lead to an increase in the stress intensity factor at the crack tip. The results of the trial, however, showed the contrary. This was done because the bonding force between crystal particles would be significantly reduced by the frost-heaving force brought on by the water-ice’s phase transition. At this point, the stress field surrounding the fracture tip was lessened overall, which caused the stress intensity factor at the crack tip to decrease. Chen et al. [37] found that the stress intensity factors of type I and type II cracks had a certain influence on the crack propagation trajectory, and the change of the stress field at the crack tip was the main factor in crack propagation. Qi et al. [38] believed that the interaction between cracks was related to the density of crack distribution. The greater the density, the stronger the interaction between them, and the lower the strength of rock failure. However, freeze-thaw erosion can exacerbate the development of interior cracks in sandstone; therefore, the more freeze-thaw cycles there were, the more internal cracks they had, and the weaker the sandstone was when it was destroyed.

## Conclusions

In the study, the effects of freeze-thaw cycles on the mechanical properties, energy evolution, full-field strain mode, and fracture development of sandstone were investigated. Based on the theory of fracture mechanics, the change in stress intensity factor at the crack tip under freeze-thaw action was analyzed. The conclusions are drawn below:

1. Sandstone’s mechanical properties declined after freeze-thaw cycles. When there had been *80* freeze-thaw cycles, the sandstone’s elastic modulus had dropped from *1*.*46 GPa* to *0*.*13 GPa*, the crack-initiation stress had dropped by *95%*, the peak stress had dropped by *92%*, and *σ*_*ci*_*/σ*_*c*_ had gradually declined as the number of freeze-thaw cycles had grown. The alteration of *σ*_*ci*_*/σ*_*c*_ revealed the opposite pattern after *60* cycles of freeze-thaw.
2. The more elastic energy that could be stored during the elastic stage, the more brittle the sandstone was. The number of freeze-thaw cycles increased, which resulted in a decrease in the total energy, elastic energy, and dissipation energy at various stress thresholds. When the number of freeze-thaw cycles approached *60*, the rate of energy reduction at different stress thresholds increased, but the rate of dissipation energy reduction at the crack initiation stress point was unaffected.
3. More unevenly distributed sandstone strain fields and bigger post-failure cracks result from higher freeze-thaw cycle counts. The axial and radial strains in both the cracked area and the non-cracked area increased along with the number of freeze-thaw cycles. The stress intensity factor at the crack tip was negatively correlated with the number of freeze-thaw cycles and positively correlated with the inclination angle of the crack tip.

## Supporting information

S1 File (DOCX)

S1 Data (ZIP)

## Abbreviations

$\bar{\varphi_{1}}$: The average of *φ*_1_
$\bar{\varphi_{2}}$: The average of *φ*_2_
$\bar{d}\left(\bar{x};\bar{a}\right)$: The displacement vector
$\bar{a}$: The parameters to determine the displacement vector in a given model
*C*(*a*): The calculated image block
*K*_1_: The stress intensity factor
U: The total energy
U_D_: The dissipative energy
U_E_: The elastic potential energy
*u*_x_: The displacement along x direction
*u*_y_: The displacement along *y* direction
*Θ*: The dip angle of crack tip
μ: Poisson ratio
σ_c_: Peak stress
σ_ci_: Initiation stress
φ_1_: The intensity of pixel $\bar{x}$
φ_2_: The intensity of pixel $\bar{x}$
