# Supplementary material for: Energy evolution and crack development characteristics of sandstone under freeze-thaw cycles by digital image correlation
Source: PLoS One. 2023 Apr 20;18(4):e0283378. doi: 10.1371/journal.pone.0283378 (PMC10118107; doi:10.1371/journal.pone.0283378)
Supplement: S1 File — (DOCX) [file pone.0283378.s001.docx]

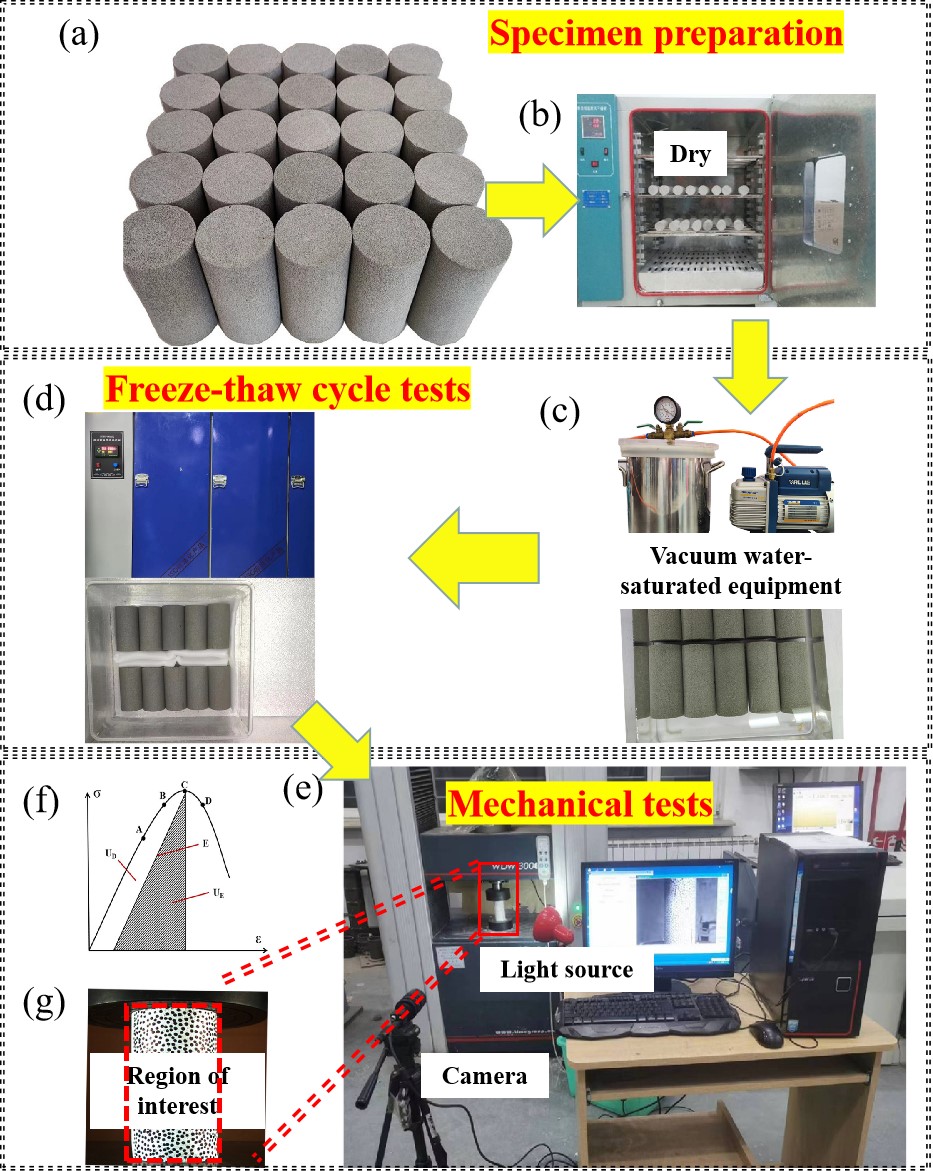


Fig. 1 Test equipment and procedure (a) Preparation of rock specimens (b) Electric drying oven (c) Vacuum water-saturation equipment (d) Freeze-thaw cycle equipment (e) Digital speckle and mechanical test (f) Energy evolution characteristics (g) Speckle specimen.

**Specimen descriptions and preparation**

Sandstone specimen adopts a kind of sedimentary rock in western Sichuan plateau. It is sound-proof, moisture-absorbing and radiation-free, and thus usually used as building material. The uniaxial compressive strength is about 40 GPa. The elasticity modulus is about 3.8 GPa. The Poisson’s ratio is 0.25 and the density is 2.4 g/cm^3^. All the specimens came from the same rock mass. It was made into cylindrical specimens with a diameter of 50 mm and a length of 100 mm, and the end surfaces of specimens were polished to ensure its flatness is less than 0.05 mm (Fig. 1a), according to International Society for Rock Mechanics (ISRM) Standard. A temperature of 105 °C was used to bake the sample (Fig. 1b), and it was weighed every 12 hours. The sample was considered to have fully dried when the difference in weight reading was less than 0.01 g. The sample was then placed in a vacuum water-saturated device (Fig. 1c) for water-saturated treatment.

According to the variation of annual average temperature under natural conditions in this area, the freezing temperature of sandstone specimen was set to -20 °C, and the melting temperature was set to 20 °C. The time of freezing and thawing was set to 12h, and the same type of sandstone was subjected to 20, 40, 60, 80 freeze-thaw cycles respectively. The freeze-thaw cycle is shown in the Fig.1d.

Load-displacement data can be directly obtained during the experiment. However，it is difficult to measure the change of local strain field of the specimen. Due to the small specimen size, sticking a strain gauge will affect the stability and accuracy of measurement. So non-contact monitoring equipment is needed for monitoring the specimen. In this study, the digital speckle system was adopted as the detection device for local strain field. The digital speckle method needs to add artificial speckle on the surface of the specimen so that the local displacement field on the surface of the specimen could be obtained. To increase the contrast ratio of the pattern, firstly, matte white paint was sprayed on the specimen surface as primer and then the black mark pen was used to draw black speckles. The process followed the principle of uniform and random distribution (Fig. 1g).


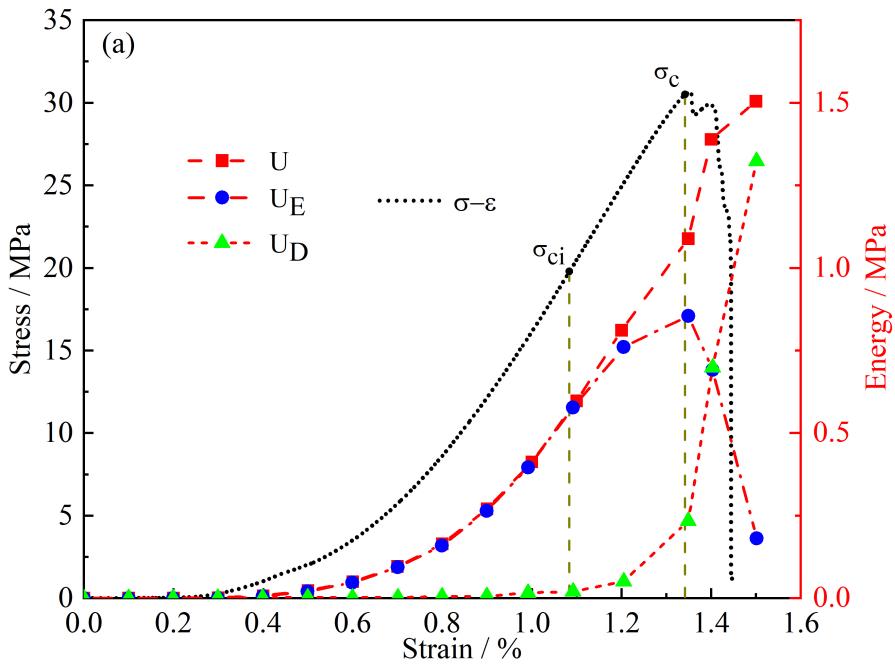

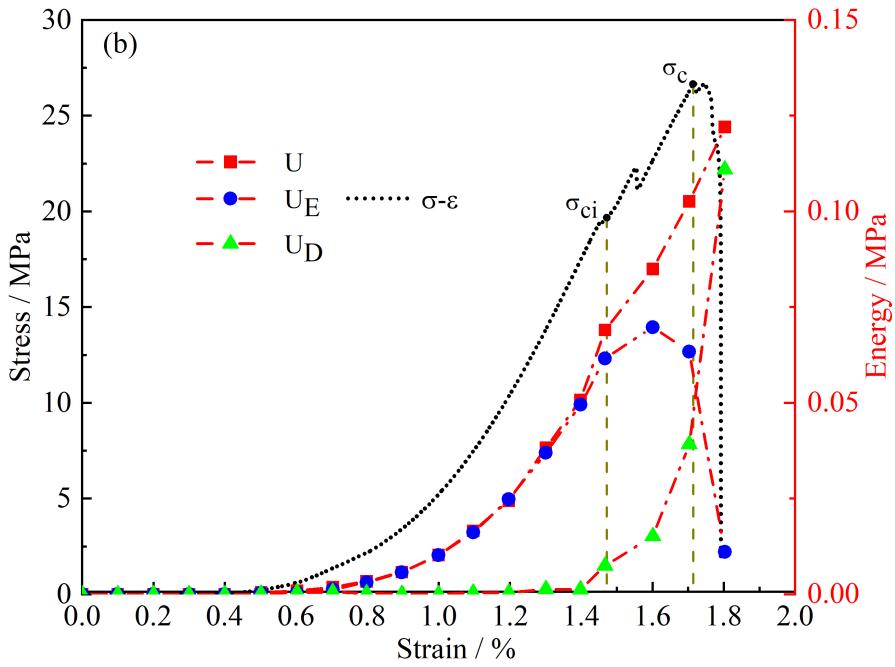

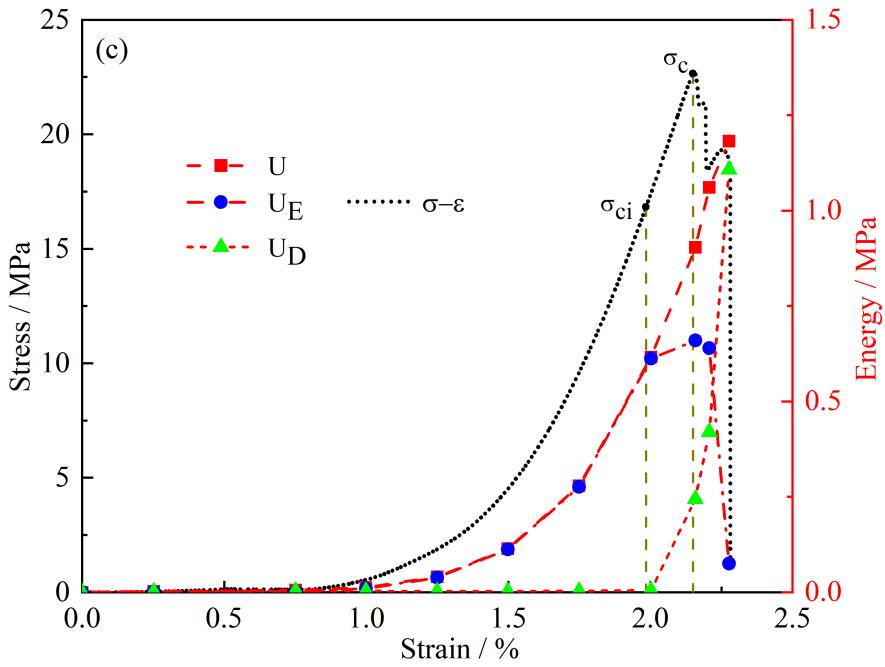

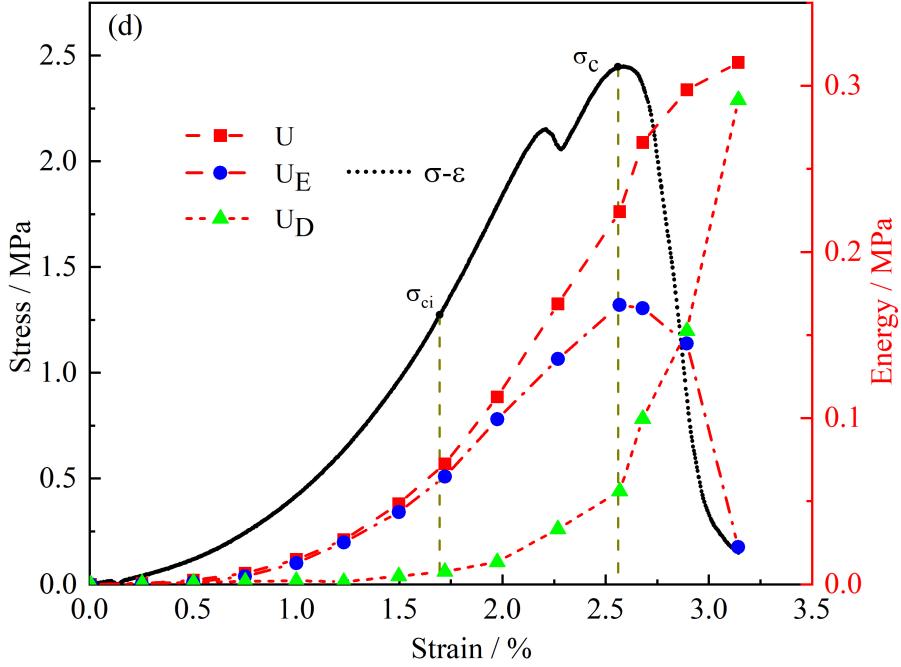


Fig. 2 Stress-strain curve and energy evolution law of sandstone with different freeze-thaw cycles (a) N=20 (b) N=40 (c) N=60 (d) N=80.

**Effect of freeze-thaw cycles on physical and mechanical parameters of sandstone**

The stress-strain curves of sandstone subjected to different freeze-thaw cycles were shown in Fig. 2. It could be found in the stress-strain diagram obtained from the uniaxial compression test that the curve had obvious characteristics in different stages. During the first stage of loading, with the increase of strain, the stress changes fluctuated, during which there was ups and downs due to the uneven distribution of pores within the sandstone. At this time, the specimen was in the compaction stage, which made the sandstone structure more compact. The curve of the second stage rose linearly, and the sandstone particles at this time were regarded as completely elastic materials. The deformation of sandstone at this stage was elastic deformation. In the third stage, the slope of the curve decreased gradually with the increase of strain, and the shape of the curve was convex. This stage was the most active stage of crack development within the specimen. Macroscopic cracks were gradually formed on the surface of the specimen with the loading of stress. In the fourth stage, the sandstone was destroyed and the curve showed a downward trend, but the specimens with different freeze-thaw cycles had different characteristics of decline, and the slope of the curve decreased gradually with the increase of freeze-thaw cycles.

Table 1 Physical and mechanical parameters of sandstone with different freeze-thaw cycles

| Freeze-thaw cycles /N | Elastic modulus  /GPa | | | Initiation stress σ_ci_  /MPa | | | Peak stress σ_c_  /MPa | | | σ_ci_/σ_c_  % | | |
| --- | --- | --- | --- | --- | --- | --- | --- | --- | --- | --- | --- | --- |
|  | Specimen | | | | | | | | | | | |
|  | 1 | 2 | 3 | 1 | 2 | 3 | 1 | 2 | 3 | 1 | 2 | 3 |
| 20 | 1.46 | 1.53 | 1.49 | 25.15 | 23.78 | 24.57 | 30.60 | 33.28 | 31.35 | 82.19 | 71.45 | 78.37 |
| 40 | 1.32 | 1.28 | 1.35 | 17.51 | 18.23 | 17.33 | 26.73 | 27.59 | 29.31 | 65.51 | 66.07 | 59.13 |
| 60 | 0.88 | 1.20 | 0.91 | 17.13 | 17.65 | 16.28 | 22.54 | 23.67 | 21.89 | 75.99 | 74.57 | 74.37 |
| 80 | 0.13 | 0.25 | 0.19 | 1.34 | 2.56 | 1.59 | 2.44 | 3.67 | 3.59 | 54.92 | 69.75 | 44.29 |

Table 1 The standard deviation of each group of data

| Freeze-thaw cycles /N | Standard deviation | | |
| --- | --- | --- | --- |
|  | Elastic modulus  /GPa | Initiation stress σ_ci_  /MPa | Peak stress σ_c_  /MPa |
| 20 | 0.035 | 0.688 | 1.383 |
| 40 | 0.035 | 0.676 | 1.314 |
| 60 | 0.077 | 0.692 | 0.901 |
| 80 | 0.060 | 0.644 | 0.688 |

Table 1 showed the changes of mechanical properties and stress thresholds of three sandstone specimens in each group. Table 2 showed the standard deviation of each group of data. The initial elastic modulus of the specimen was 3.8 GPa. As the freeze-thaw cycle test progressed, the elastic modulus of the specimens after 20, 40, 60 and 80 freeze-thaw cycles decreased by about 62% , 65% ,77% and 97% , respectively. With the increase of freeze-thaw cycles, the elastic modulus of sandstone specimens decreased, and it was close to complete failure when freeze-thaw cycles reached 80 times.


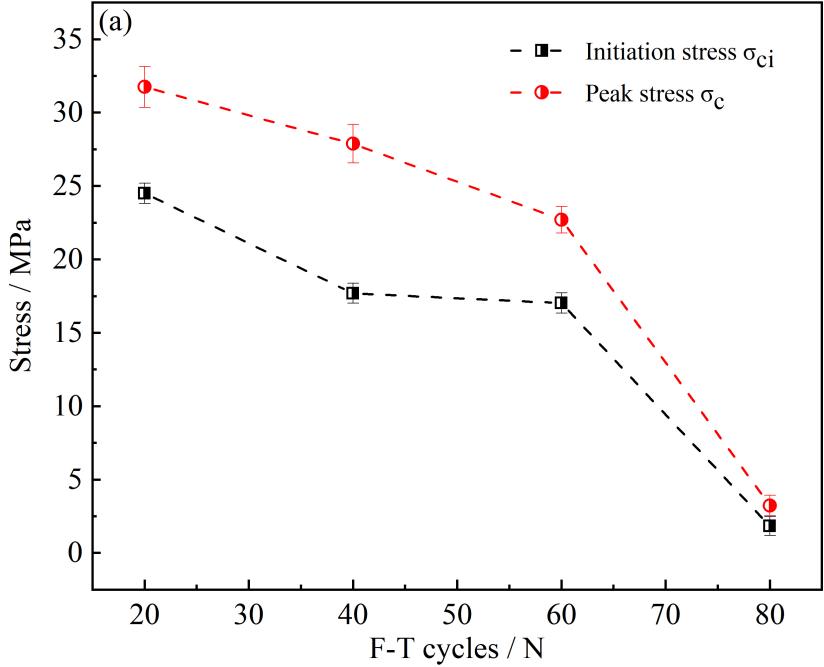


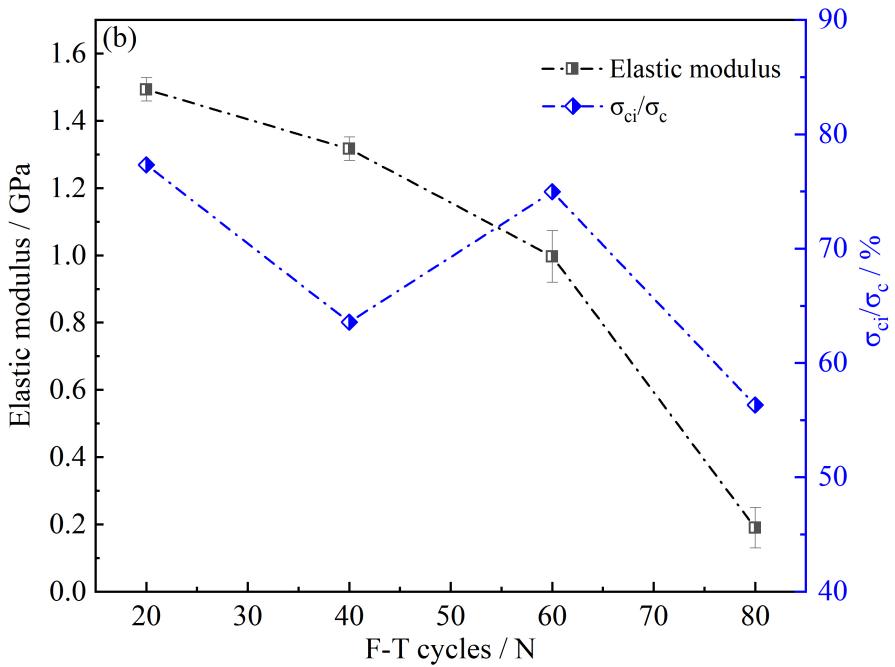


Fig. 3 The stress threshold of sandstone specimens under different conditions

As shown in Fig. 3, with the increased of freeze-thaw cycles, the initiation stress and peak stress decreased. The average value of crack initiation stress decreased from 24.5 MPa (N = 20) to 1.8 MPa (N = 80), which was reduced by 92.5%. The average value of peak stress decreased from 31.6 MPa (N = 20) to 3.2 MPa (N = 80), which was reduced by 89.9%. The decreasing values of the two were basically similar, but the ratio of initiation stress to peak stress gradually decreased with the increase of freeze-thaw cycles, from 77.3% (N = 20) to 56.3% (N = 80).

**Effect of freeze-thaw cycles on energy evolution of sandstone**

Fig. 2 showed the energy evolution trend of sandstone specimens with different freeze-thaw cycles. The evolution of total energy, elastic energy and dissipative energy was less affected by freeze-thaw cycles. The total energy of the sandstone increased nonlinearly. In the pre-peak stage of the stress-strain curve, the elastic energy showed an upward trend, the growth rate was the fastest in the elastic stage, and the elastic energy gradually decreased in the post-peak stage of the stress-strain curve. The dissipation energy always increased in a nonlinear way, and the growth rate reached its highest when the sample reached its peak stress.


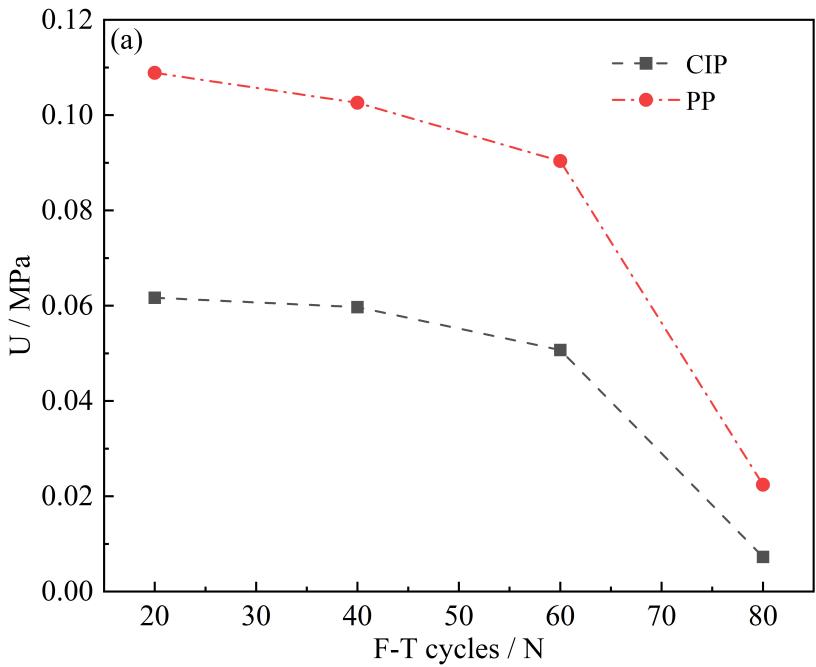

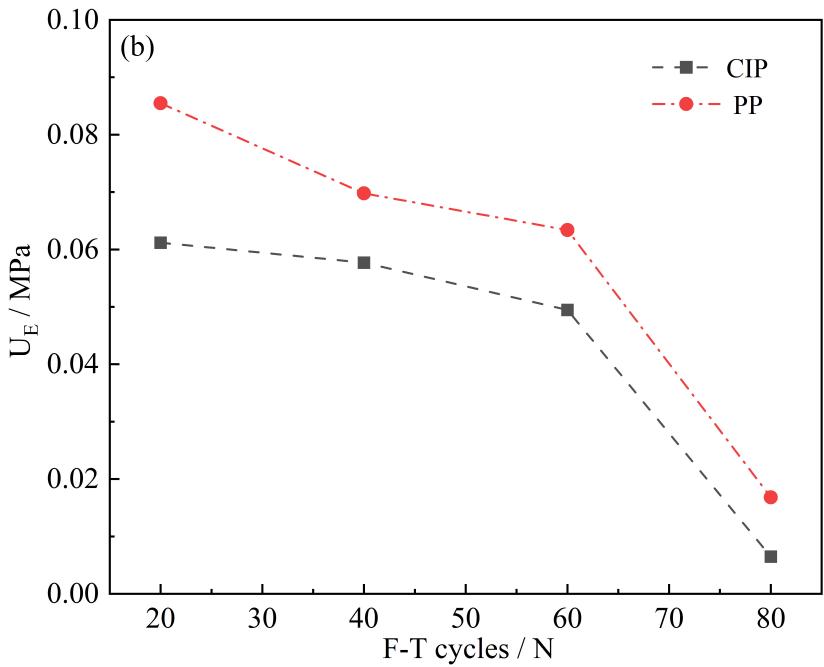


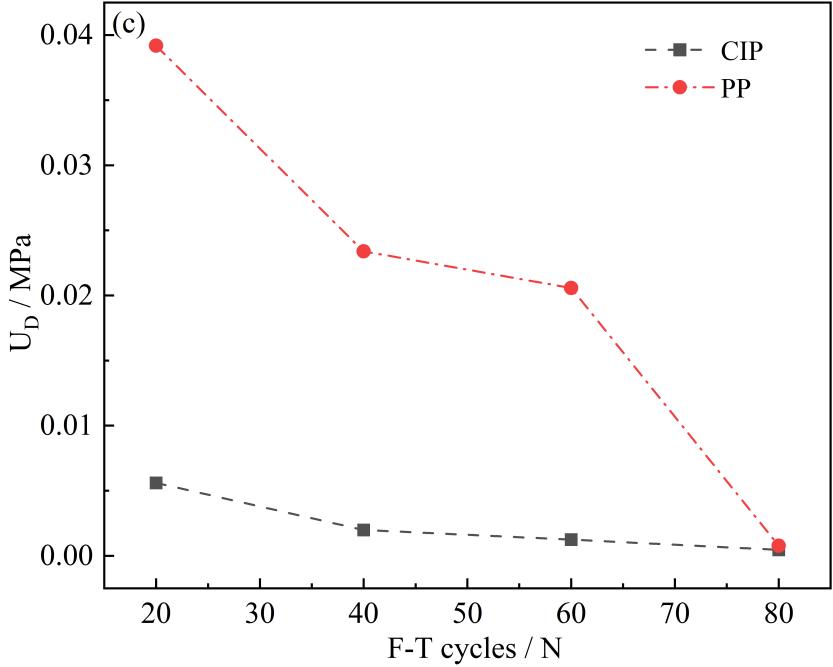


Fig. 4 F-T effects on energy at various characteristic points (a) T otal energy (b) Elastic energy (c) Dissipative energy.

The energy at various stress thresholds decreased as the number of freeze-thaw cycles increased, but the rate of decline varied (Fig. 4). CIP represents Crack initiation Point. PP represents Peak Point. Except for the dissipation energy curve at the fracture initiation stress point, each curve had clear inflection points once the number of freeze-thaw cycles reached 60.


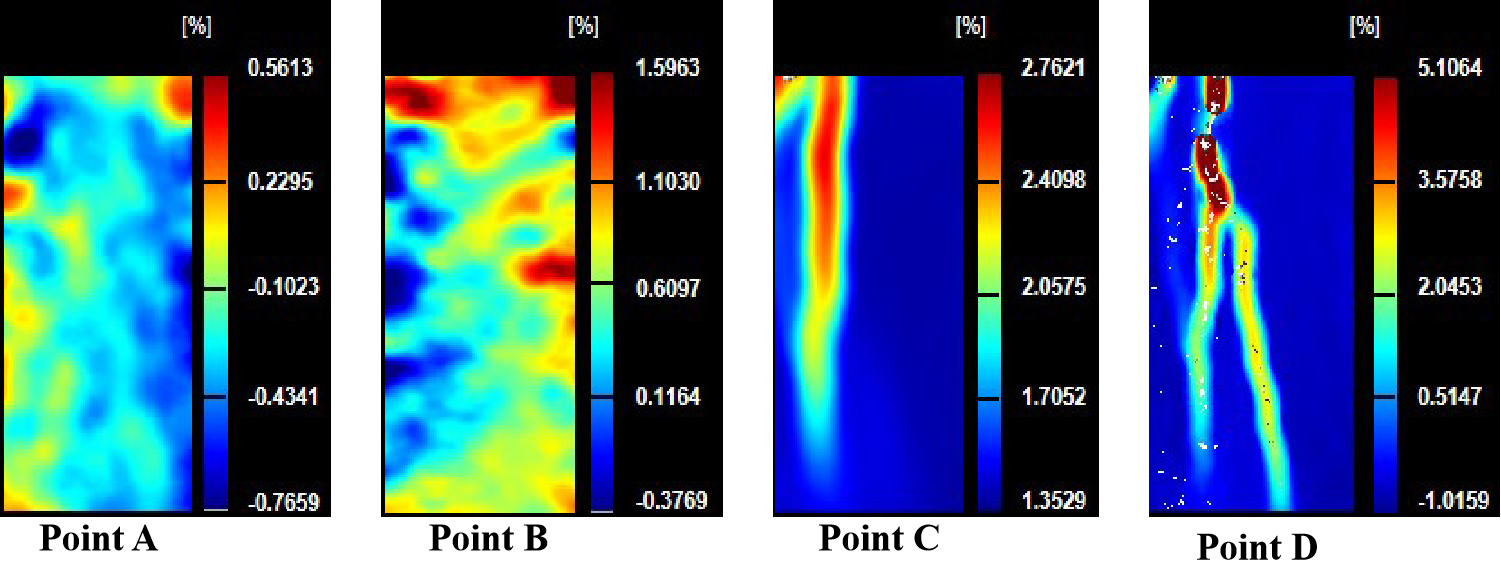


(a)


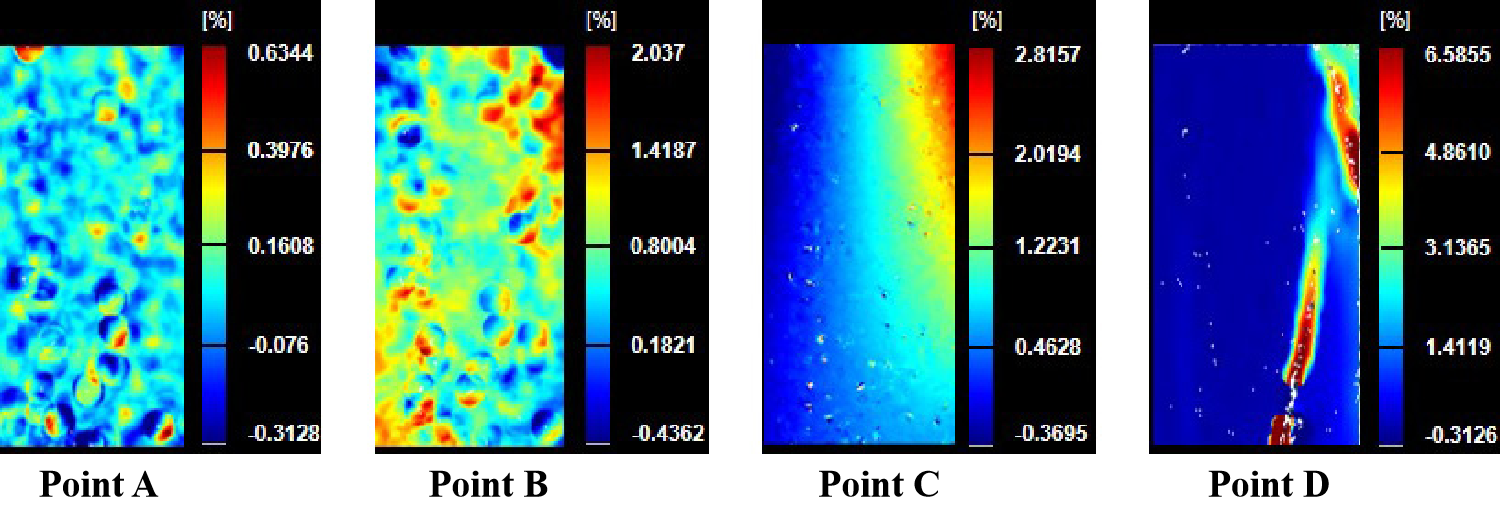


(b)


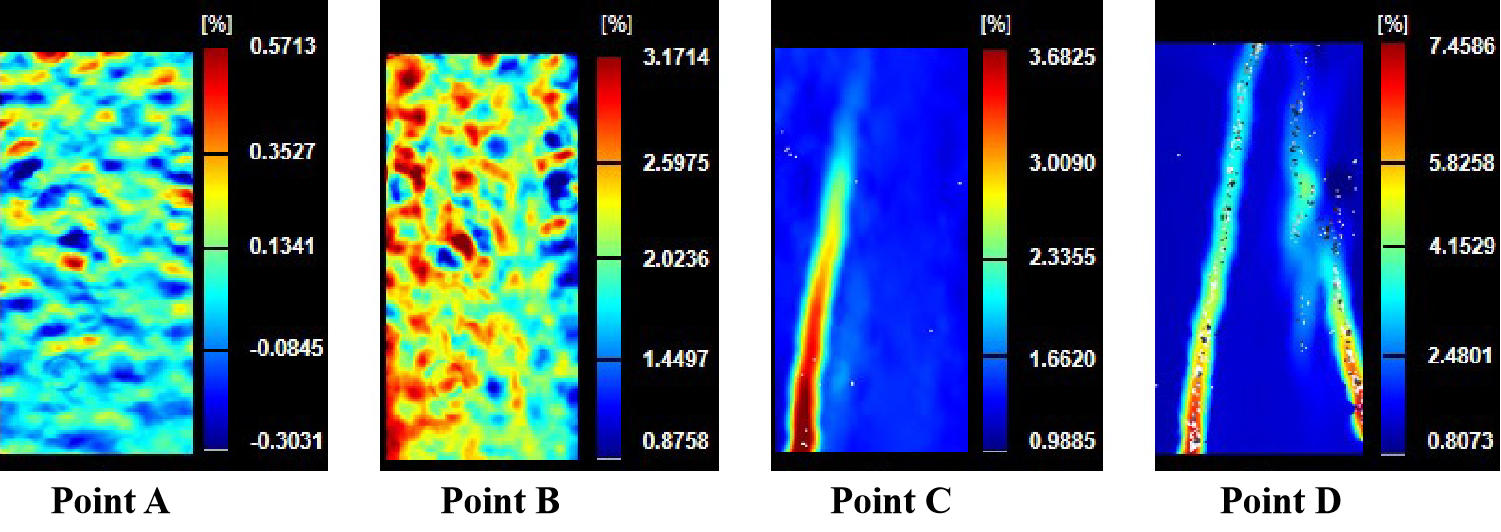


(c)


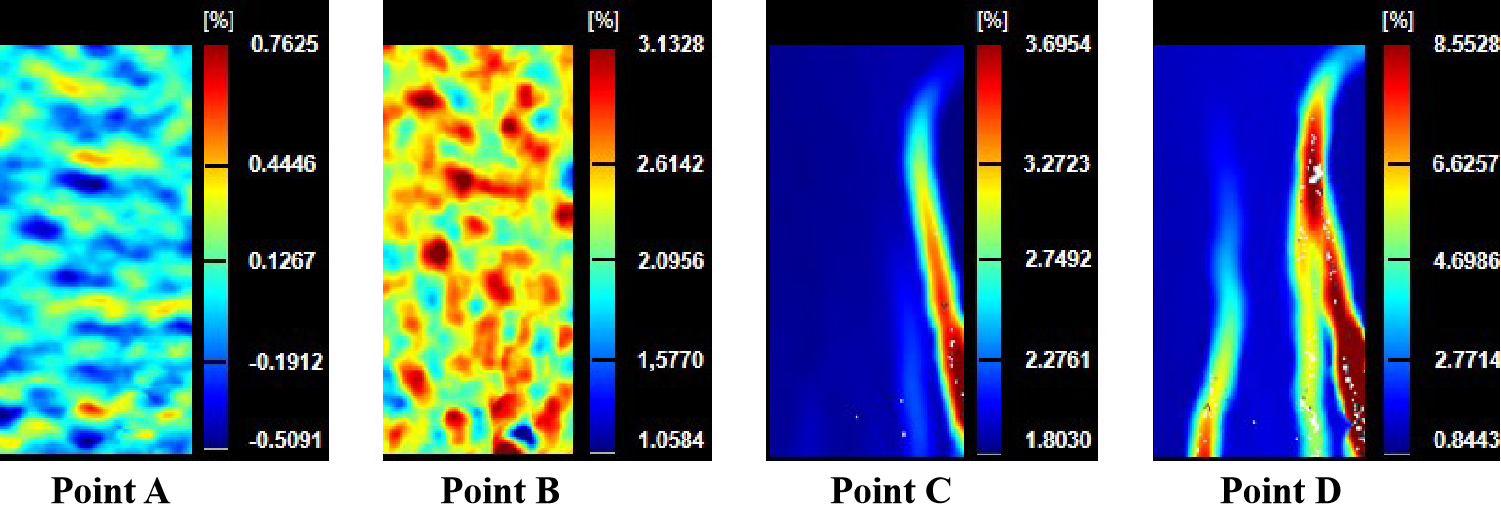


(d)

Fig. 5 Local strain field evolution of sandstone with different freeze-thaw cycles (a) N=20 (b) N=40 (c) N=60 (d) N=80.

**Local strain field and crack evolution characteristics of sandstone with different freeze-thaw cycles**

Fig. 5 showed the evolution process of the principal strain field of sandstone specimen with different freeze-thaw cycles under uniaxial compression conditions obtained by digital image correlation technology (DIC). In different loading stages, the strain field on the specimen surface had different characteristics. At point A in the pre-peak stage, the main strain field was uniformly distributed, and the color of the nephogram was close to the monochromatic pattern, but there were also some unique color areas on the surface of the sample. In the image captured at point B of the stress-strain curve, areas with unique and obvious colors began to extend on the original basis, and new areas with unique colors were continuously generated at other positions on the sample surface. At the point C of the stress-strain curve, many small areas with similar directions produced strains that converge on the surface of the sample, and the outline of the main crack was completely presented, and as the loading progressed, the main crack continued to expand. At the D point of the stress-strain curve, the main crack had reached the maximum degree of expansion and extension, and the surface of the specimen had developed derived cracks.


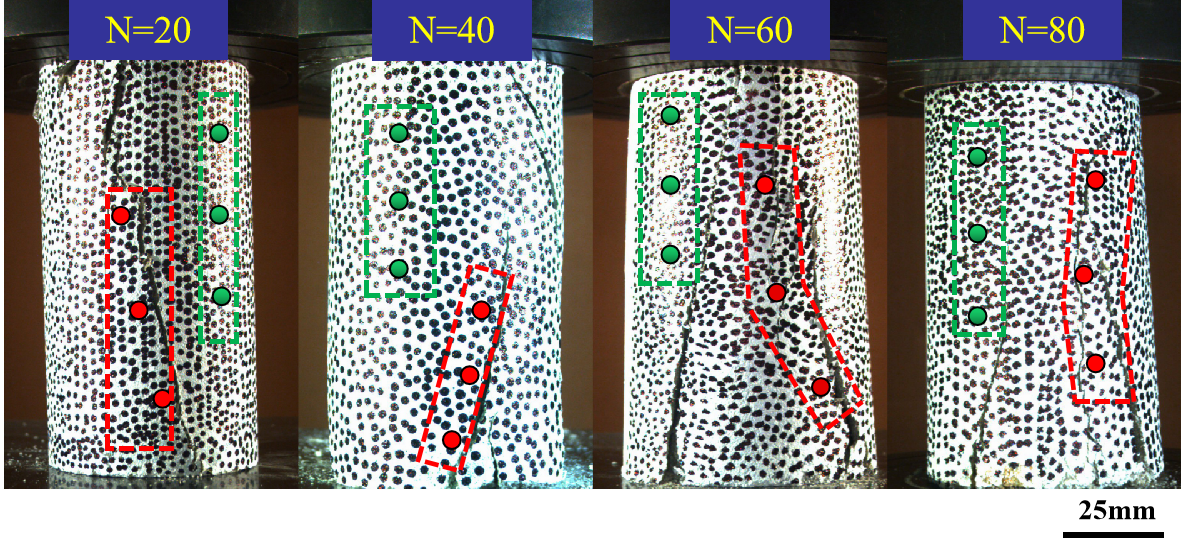
Fig. 6 Location of monitoring points in the cracked zone and the non-cracked zone.

**Strain characteristd area and non-cracked area**

In order to quantitatively analyze the strain field of cracked area and non-cracked area, three monitoring points were set up in cracked area and non-cracked area respectively. The red point is the monitoring point of the cracked zone; Green point is the monitoring point of non-cracked zone, as shown in Fig. 6..


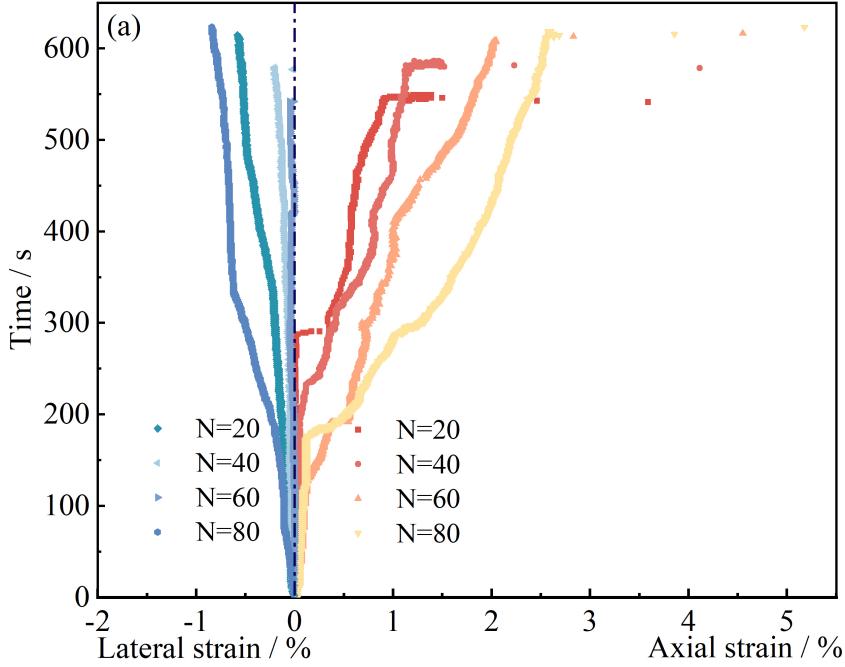


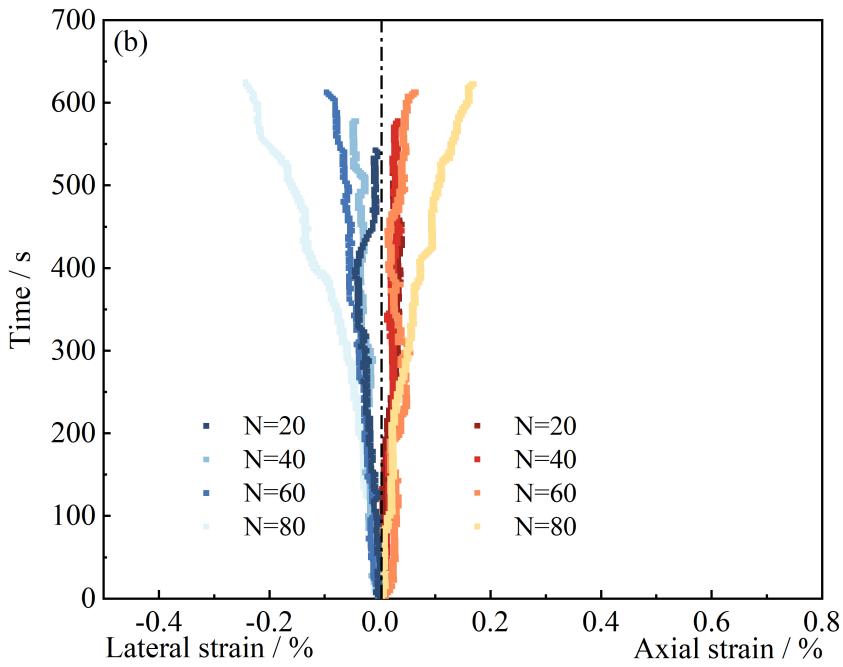


Fig. 7 The variation of strain with time (a) Strain in cracked area (b) Strain in non-cracked area.

As shown in Fig. 7a, the local strain in the cracked area was obvious at about 294 s for the sample with 20 freeze-thaw cycles, and with the increase of freeze-thaw cycles, the significant local axial strain appeared earlier. When the number of freeze-thaw cycles reached 80, the sample produced significant local axial strain at about 176 s. When the progressive failure inside the specimen accumulated to a certain extent, significant local axial strain began to appear on the surface of the specimen. As the loading progressed, the strain on the monitoring point in the cracked area gradually increased. The specimen with 20 freeze-thaw cycles was mainly composed of tensile cracks. With the increased of freeze-thaw cycles, the monitoring points around the cracked area showed an obvious shear slip phenomenon. The radial strain of the cracked area increased with the increase of freeze-thaw cycles, and the growth trend was close to linear growth.

**Effect of freeze-thaw cycles on the stress intensity factor**


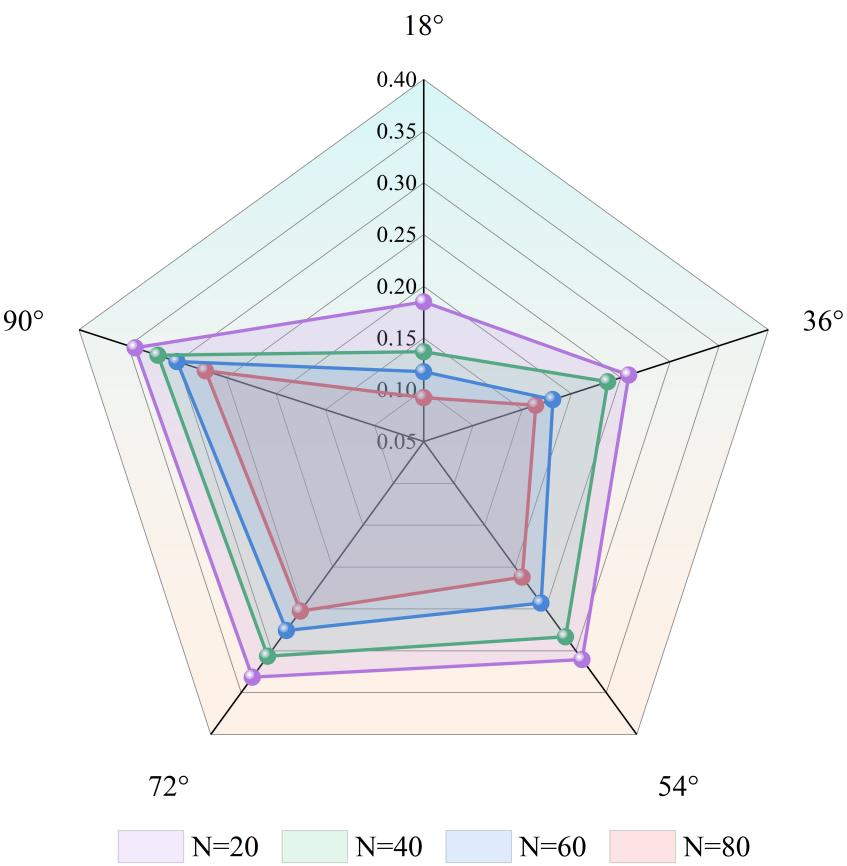


Fig. 8The variation law of the stress intensity factor at the crack tip

As shown in Fig. 8. Under the same freeze-thaw cycle conditions, the stress intensity factor at the crack tip gradually increased with increasing crack dip angle. The stress intensity factor at the crack tip decreased as the number of freeze-thaw cycles increased. The tension near the crack tip and the crack length were all positively connected with the stress intensity factor.
